# Theoretical and experimental assessment of degenerate primer tagging in ultra-deep applications of next-generation sequencing

**DOI:** 10.1093/nar/gku355

**Published:** 2014-05-07

**Authors:** Richard H. Liang, Theresa Mo, Winnie Dong, Guinevere Q. Lee, Luke C. Swenson, Rosemary M. McCloskey, Conan K. Woods, Chanson J. Brumme, Cynthia K.Y. Ho, Janke Schinkel, Jeffrey B. Joy, P. Richard Harrigan, Art F.Y. Poon

**Affiliations:** 1 BC Centre for Excellence in HIV/AIDS, Vancouver, BC, V6Z 1Y6, Canada; 2 Section of Clinical Virology, Department of Medical Microbiology, Academic Medical Center, 1105AZ Amsterdam, The Netherlands; 3 Department of Medicine, University of British Columbia, Vancouver, BC, V5Z 1M9, Canada

## Abstract

*Primer IDs* (*pIDs*) are random oligonucleotide tags used in next-generation sequencing to identify sequences that originate from the same template. These tags are produced by degenerate primers during the reverse transcription of RNA molecules into cDNA. The use of pIDs helps to track the number of RNA molecules carried through amplification and sequencing, and allows resolution of inconsistencies between reads sharing a pID. Three potential issues complicate the above applications. First, multiple cDNAs may share a pID by chance; we found that while preventing any cDNAs from sharing a pID may be unfeasible, it is still practical to limit the number of these collisions. Secondly, a pID must be observed in at least three sequences to allow error correction; as such, pIDs observed only one or two times must be rejected. If the sequencing product contains copies from a high number of RT templates but produces few reads, our findings indicate that rejecting such pIDs will discard a great deal of data. Thirdly, the use of pIDs could influence amplification and sequencing. We examined the effects of several intrinsic and extrinsic factors on sequencing reads at both the individual and ensemble level.

## INTRODUCTION

In population sequencing applications, Sanger sequencing by the detection of chain-terminating dideoxynucleotides can only reproducibly detect minority variants that comprise a minimum of about 20% of the template population (1–5). Next-generation sequencing (NGS) platforms can provide considerably greater sensitivity because they use large-scale parallelization of sequencing reactions to automate the sequencing of thousands of individual templates at once. The high throughput afforded by NGS comes, however, at the cost of higher rates of sequencing error relative to Sanger sequencing. In addition, the frequency of variants among sequences (reads) produced by NGS does not necessarily reflect their respective frequencies in the population of templates because of the inherently stochastic nature of polymerase chain reaction (PCR) amplification. While this resampling error affects all PCR-based methods of sequencing, the increased sensitivity of NGS is more susceptible to this effect.

Primer IDs (pIDs) are ‘tags’ used in NGS to address these problems (6,7). pIDs are produced by incorporating a string of degenerate nucleotides into the cDNA synthesis primer. These produce a random string of nucleotides that appears in each cDNA molecule produced by reverse transcription from an original RNA molecule. After PCR amplification, this random label thus appears in all of the produced DNA copies of the original. Each pID consists of a string of random nucleotides appearing somewhere (typically in the primer) in each sequenced observation coming from the NGS platform. In principle, these oligonucleotides function to identify all DNA copies after PCR amplification that were derived from the same RNA molecule. This not only enables the investigator to estimate the number of RNA molecules being represented by the reads produced by NGS, but also provides a means of error correction by averaging out variation due to sequencing error among reads with a common pID. We use the term ‘pID design’ to refer to the number, type and arrangement of degenerate nucleotides that will determine the composition of pIDs. There are two major considerations in pID design: first, the number of degenerate nucleotides or length of the pID; and secondly, what combination of fully or partially degenerate nucleotides will make up this number, where a partially degenerate nucleotide can represent either two or three of the nucleotides. Together, these factors determine the maximum number of pIDs; for example, a design of the form NNNRNNN (using IUPAC symbols for degenerate nucleotides) admits 4^6^ × 2 = 8192 possible pIDs. In this study, we use analytical and experimental methods to evaluate the practical consequences of pID design in the context of characterizing a population of RNA molecules by NGS-based resequencing or ‘deep sequencing’. We consider three distinct but interrelated consequences of pID design.

First, there is always a chance that two RNA molecules receive the same pID (a *collision*), the probability of which is determined by the number of pIDs and the number of RNA molecules going into the reverse transcription and amplification reactions. If this happens, the two RNA molecules can no longer be distinguished using the pID. Assuming that all possible pIDs are equally likely to attach to any given RNA molecule, this situation is known in probability theory and statistics as the *birthday problem* (8). We carry out numerical analyses of the probabilities of attaining perfect or ‘adequate’ labelling of RNA molecules by pIDs under varying conditions, where labelling is considered adequate when the vast majority of RNA molecules receive unique pIDs. Our analysis indicates that adequate labelling is considerably easier to achieve than perfect labelling under realistic experimental conditions, while retaining the advantages of perfect labelling.

Secondly, shallow *read depth* of RNA molecules, or number of reads (i.e. deep-sequencing observations) coming from a given RNA molecule, is another effect of pID design. As discussed previously, another benefit to using pIDs is the ability to turn resampling or oversampling to our advantage by using it to correct for sequencing error. If the amplicon pool has been perfectly labelled, or well-enough labelled that any collisions in labelling are infrequent enough to not significantly affect the data, then one can exactly trace which reads correspond to the same original RNA molecule. In order to construct a consensus sequence for an RNA molecule, there must be at least three reads for that molecule, as ties between two reads cannot be resolved. (In fact, three or more reads may still create such ties—for example if three reads have A, C and G at a given base—but this is considered to occur infrequently enough to be a negligible possibility.) Because of this, it is suggested that RNA molecules with read depth below three be rejected from analysis as a more trustworthy consensus sequence cannot be formed (6). To assess the impact of shallow read depths, we perform serial dilution and Roche 454 NGS experiments on two plasma samples from HIV-positive subjects. In addition, we analyse data from another 454 NGS experiment on serum samples serially obtained from three hepatitis C virus (HCV)-positive subjects. We find that substantial fractions of data are unavailable for error correction because they are represented by fewer than three reads. Using these data, we develop a generalized statistical model of the sensitivity of shallow read depth to experimental conditions.

Thirdly, we consider whether using pIDs introduces any biases to the resulting data. If using pIDs might cause a certain sequence variant to be observed in numbers completely unlike its actual prevalence, then we cannot trust the results obtained using this technique. Using data from the same experiments, we examine the effects of the different pIDs on amplification/sequencing bias. Since the 454 platform is particularly susceptible to insertion/deletion (indel) errors when sequencing homopolymers, and since pIDs can introduce homopolymers to the sequenced DNA molecules, we also examine the effects of pIDs on indel error probabilities at the microscopic level.

Overall, we find that the pID technique is accompanied with several intrinsically unavoidable challenges. However, our models and experimental results indicate that under an optimal range of experimental conditions, some of these challenges, such as imperfect labelling, may have a minimal impact on the utility of this technique. We also find that the other challenges can be overcome with careful consideration of experimental design and by more recent advances in NGS technologies that can yield much higher numbers of reads. The principles behind the pID method and theoretical analysis performed here apply generally to sequencing of an RNA population (or DNA population sequencing, though the wet bench procedures we describe would not apply). Our primary experiment consists of HIV viral population sequencing on the 454 platform; the technique was originally developed in this context (6), and it has been of great interest to researchers and clinical practitioners in this field. We also evaluate the pID method on NGS data derived from HCV populations using the 454 platform under a different set of experimental conditions. We describe these analyses and experiments in the following.

## MATERIALS AND METHODS

To avoid confusion between the different types of products and molecules occurring at different stages of the process of reverse transcription, amplification and sequencing, we will introduce the following terminology. (These terms are introduced in the context of HIV sequencing, but they also apply to the HCV case.) In HIV RNA sequencing, one starts with a plasma *sample*; from this sample, an *extract* containing RNA molecules is produced; from this extract, a portion of the RNA molecules is reverse transcribed into complementary DNA (cDNA) and amplified via PCR to produce an *amplicon pool* consisting of DNA copies (or *amplicons*) of the cDNA. The HCV experiment followed the same steps except that the starting samples were serum samples rather than plasma samples. The same principles hold for any other RNA sequencing procedure which follows these steps.

We define the *RNA molecule count* of an amplicon pool to be the total number of HIV RNA molecules that are represented by DNA copies in the pool after extraction, reverse transcription and amplification. Neither extraction nor reverse transcription is completely efficient, and if an RNA molecule is lost during extraction or fails to reverse-transcribe, then this RNA molecule will never be amplified and, therefore, never sequenced. These inefficiencies must be considered when estimating the RNA molecule count of an amplicon pool.

Another piece of convenient notation: we will refer to a pID design consisting of *m* consecutive N's as N*m*. For example, the design NNNNNNNN is N8. For a given design length *m*, N*m* is the design that admits the most possible pIDs.

*Labelling*: Let us briefly describe the birthday problem and its effect. Assuming that all possible pIDs are equally likely to bind to any RNA molecule during the application of sequencing primers to the RNA molecules, the probability that *n* RNA molecules all receive unique pIDs from a pool of *N* possible choices is:

(1) \begin{equation*} \prod _{i=0}^{n-1} \frac{N-i}{N} = 1\times \frac{N-1}{N}\times \cdots \times \frac{N-n+1}{N}. \end{equation*}

We call such an event *perfect labelling*. This ignores the fact that not all RNA molecules in the sample population have been extracted and reverse transcribed, meaning that there are ‘lost’ RNA molecules that are never tagged or amplified, and, therefore, never represented in the eventual sequencing product. However, assuming further that all RNA molecules are independently equally likely to be lost, the same formula holds if *n* is taken to be the number of RNA molecules that successfully reverse transcribe and *N* is the same pool of possible pIDs.

Computing the number of pIDs required to achieve a high probability of ‘good enough’ coverage (under the continuing assumption that all possible pIDs are equally likely to attach to any given RNA molecule) is a combinatorial problem: the formulae are reasonably straightforward to derive, but are computationally difficult. For example, suppose that we would like to compute the probability that all but 5 RNA molecules receive unique pIDs. The problem is similar in spirit to the classic problem of counting the number of ways to assemble a poker hand, such as two pair or three of a kind. We need to enumerate all the possible ways that we could end up with five RNA molecules sharing pIDs, compute the probabilities of each and sum them. Is there one pair of RNA molecules sharing one pID and another three sharing a second pID, for example, or do five RNA molecules all share one pID? We use the resulting formulae to compute the number of possible pIDs required to limit the number of collisions in a manner we make precise below.

Let us introduce some notation to assist in the counting of certain assignments of pIDs to RNA molecules. We say that a pID is a *k-tag* if it is shared by *k* RNA molecules. For example, if perfect labelling is achieved for an amplicon pool representing *n* RNA molecules, then there are exactly *n* 1-tags. If there are two pIDs that get assigned two RNA molecules each, one that gets assigned three RNA molecules, and then the rest of the RNA molecules get unique pIDs, then this amplicon pool has one 3-tag, two 2-tags and (*n* − 7) 1-tags.

We define a probabilistic model to describe the labelling of RNA molecules with pIDs and use combinatorial probability calculations (described in Section S2.2 of the Supplementary Materials) to compute the probabilities for ‘95% good’ labelling, which we define as consisting of outcomes where the number of distinct pIDs represented in the amplicon pool is at least 95% the number of RNA molecules. (Note that this guarantees that at least 90% of the RNA molecules represented in the amplicon pool have unique pIDs; in this worst-case scenario, the other 10% have all collided and received pIDs that they share with one other of these unlucky molecules.) This accepts that there will likely be pIDs shared by several RNA molecules represented in our amplicon pool, but in small quantities. When this becomes too computationally expensive to perform (for large RNA molecule counts), we continue on with two methods. First, we compute these probabilities for labellings better than 95% good, which reduces the amount of computation necessary because, in the language of Section S2.2 of the Supplementary Materials, fewer *k*-tag configurations need to be considered. Secondly, to get results for 95% good labelling of larger RNA molecule counts, we compute approximations as described in Section S2.3 of the Supplementary Materials.

### Shallow read depth

In the following, we define a *k-RNA* to be an RNA molecule with read depth *k*. We will call 1- and 2-RNAs *singletons* and *doubletons*, respectively.

Our primary HIV data are drawn from a serial dilution experiment using plasma samples coming from two HIV-positive subjects: P1, reported viral load (rVL) 3.12 × 10^5^ copies/ml; and P2, rVL 1.78 × 10^5^ copies/ml. (We make a distinction between *reported* viral load and true pVL to emphasize the error occurring in the viral load assay.) For each, 60 μl of eluent is produced from 0.5ml of plasma. Each plasma extract is serially diluted so that extracts at full concentration, 10-fold dilution, and 100-fold dilution were considered.

Each of the 2 × 3 = 6 resulting subject-dilution combinations is amplified starting with 5μl of extract, targeting codons 96–194 of the HIV reverse transcriptase (HIV-specific primer sequence TTTGYTCTATGCTGCCCTAT; note that the degenerate nucleotide Y is not used as part of a pID, but because both C and T frequently appear in that position of HIV sequences), using different degenerate primers representing different pID designs. The pID designs considered are: N9 (4^9^ = 262144 possible pIDs); NNDNNHNNV (4^6^ × 3^3^ = 110592 possible pIDs) and NBDHVBDHV (4 × 3^8^ = 26 244 possible pIDs).

Three replicates are performed for each of the 2 × 3 × 3 = 18 subject-dilution-design combinations; we refer to a subject-dilution-design-replicate combination as a *batch*. We produce 2 × 3 × 3 × 3 = 54 batches as above. In addition, one replicate is produced for each subject at 10-fold dilution with a pID design of RYRYRYRYR (2^9^ = 512 possible pIDs). In total, 56 batches are so produced, and of these 54 successfully produce amplicon pools, with two failing to amplify. We will also refer to these amplicon pools simply as batches. Details of the wet bench methodology, including primers and thermal cycler settings, are included in the Supplemental Materials, Section S1.

Every batch that successfully produces an amplicon pool is sequenced on a Roche/454 GS-FLX pyrosequencing platform in the ‘reverse’ direction (from the 3′ end to the 5′ end). The sequencing plate is divided into eight sequencing regions, and batches are distributed among the first seven of them. To distinguish between batches run on the same region, a different sequencing *barcode* is attached to the cDNA molecules produced at the reverse transcription step for each batch, allowing reads to be traced back to their source batches. There are 12 such barcodes (labelled A through L), allowing 12 batches to be run on the same sequencing region. After sequencing, the data are processed using the standard 454 quality filters (9) and Python scripts that collect and collate information about the pIDs; the procedure is detailed in Section S3.1.1 of the Supplementary Materials.

We supplement this experiment with HCV viral sequencing data examining the NS5B gene taken by the 454 platform. These data are produced from serum samples serially obtained from three HCV-positive subjects, which we refer to as HCV1 (7 time points), HCV2 (14 time points) and HCV3 (11 time points). After extraction, reverse transcription and PCR amplification is performed on an amount of extract intended to contain 10 000 RNA molecules. The pID design N9 is used in all cases. The resulting data is then processed through a different processing pipeline, developed in-house. The pipeline used for our HIV experiment is built solely for that experiment, and would require modification to work for the purposes of the larger HCV experiment our data are drawn from. The pipeline used for the HCV data is newer and more general, but its raw results appear similar to those of the original pipeline (see Section S3.1.1 of the Supplemental Materials). See Sections S1 and S3.1.2 of the Supplementary Materials for further details on the experiment and the pipeline. For this experiment, we use the term ‘batch’ to refer to a specific subject and time point, and the amplicon pool coming from the corresponding sample; there are 32 batches in total.

To evaluate the relationship between the number of RNA molecules observed and the dilution of the batches, we turn to a statistical model (described in Section S3.2 of the Supplementary Materials), validating the model by comparing its predictions with both our HIV data and our HCV data. Note that our model only produces loose confidence intervals, and as such we do not claim our validations against experimental data to be particularly strong evidence in favour of our model. Nonetheless they provide a simple guide to suggest how our model performs.

### Biases at the microscopic level

We define the read depth ‘success’ of a pID in a batch to be its read depth divided by the total number of reads produced from the batch it belonged to. We examine each pID sequenced to see if its success depends significantly on several *intrinsic* factors (including the *homopolymer score*, which summarizes the extent of mononucleotide repeats present in the pID) and *extrinsic* factors, described in Section S4.1 of the Supplemental Materials. Using these explanatory variables, we fit a generalized linear model using a gamma distribution with inverse link function to our HIV data. We also perform this regression with our HCV data excluding the extrinsic factors, which are not applicable due to the differences in experimental conditions and processing pipelines.

Additionally, we analyse the susceptibility of different pIDs to be affected by indel error. An observed pID is considered to have an indel error if it is of length 8 or 10 and can be ‘corrected’ by a single insertion or deletion, respectively, to another observed pID of length 9. We perform a logistic regression on the probability that a pID is sequenced with an indel error using the same intrinsic and extrinsic explanatory factors as our regression on pID success. We restrict our data to pIDs that were observed more than once, because our definition of an indel error does not include pIDs exhibiting indel error that only appear once; they must be observed at least one time in their proper length 9 form, and at least one other time in an erroneous length 8 or 10 form. This analysis is only applicable to our HIV data; we cannot directly perform this analysis on our HCV data as the pipeline used does not perform this correction.

### Biases at the macroscopic level

We evaluated the impact of macroscopic factors on the HIV experiments where these factors were varied. Restricting to one of P1 and P2 at a time, we consider all of the sequenced batches, rejecting those that produced fewer than 500 reads (assuming such low-depth batches are indicative of a systematic problem at some point in the amplification and/or sequencing). Our data collection scheme uses the pIDs to correct for oversampling, taking the consensus of each pID's associated reads as the sequence of its corresponding RNA molecule, but we do not discard the singleton and doubleton reads. However, we do discard data coming from pIDs whose consensus sequence contained a mixture.

Focusing on a 100 bp portion of RT, for each subject we identify all variants observed in any batch coming from that subject, across all experimental conditions. For the variants occurring at higher than 2% prevalence in total, we track their prevalence within each batch to see if the pID design used or dilution of the batch have any biasing effects. There were six such variants in subject P1 and three in subject P2.

Since a visual inspection of these prevalences may not reveal any associations to the naked eye, we obtain a quantitative interpretation of these associations by performing a multinomial logistic regression on the probabilities of any given read coming from a P1 batch belonging to one of these six top P1 variants (and implicitly the probability of it belonging to any other variant outside these six), and similarly for the three top P2 variants. The explanatory factors are the pID design used and the dilution of the batch.

Using the same data, we also vary the error correction method used to examine whether sequence diversity was affected. We consider two facets of the error correction method: one, which pIDs are retained and which are rejected based on read depth (we call this the *keep scheme*); and two, how mixtures in the resulting consensus sequence are handled (we call this the *mixture resolution scheme*). The keep schemes we consider are: not rejecting any pIDs; rejecting singletons and rejecting singletons and doubletons. The mixture resolution schemes we consider are: rejecting pIDs whose consensus sequence contain any mixtures (we call this *censoring*); and counting any such pIDs fractionally towards each of the consensus sequence's possible resolutions equally (we call this *equal weighting*). For example, under this scheme the sequence AARY counts as 1/4 of a read towards all of AAAC, AAAT, AAGC and AAGT.

As our baseline, we use a keep scheme that does not reject any pIDs and censored mixtures. We compare this baseline against the other two keep schemes where singletons are rejected and where both singletons and doubletons are rejected, still censoring. Then, we compare the baseline against an error correction method using the same keep scheme (keeping all pIDs) but using an equal weighting mixture resolution scheme instead. We perform two multinomial regressions on the probabilities of a read belonging to any of the aforementioned top six P1 variants using first the keep scheme, and then the mixture resolution scheme, as the explanatory variable, and similarly for the top three P2 variants.

The HCV data do not lend itself to this analysis. All samples come from different virus populations—even the samples coming from the same subject come from different times, so that the virus population will have evolved between sampling times—so variants should not necessarily be expected to appear across samples in similar prevalences.

## RESULTS

### Perfect and 95%-good labelling

Recall that using pIDs to label RNA molecules in an NGS experiment should enable one to enumerate the number of RNA molecules represented within the resulting reads; however, if different RNA molecules receive the same labels, then this ability is reduced.

Using Equation (1) to compute the number of pIDs required to achieve a high probability (0.9) of perfect labelling for selected RNA molecule count values (see Section S2.1, and particularly Table S5, of the Supplementary Materials) shows that even for low-to-moderate reported RNA molecule counts, the number of pIDs required to assure perfect labelling may be prohibitively high. For example, the design N15 admits 1 073 741 824 possible pIDs; this is already a long design, and yet this would still be insufficient to handle amplicon pools with a reported RNA molecule count of 10 000 with an acceptable probability of perfect labelling. Let $\mathbb {P}A$$\mathbb {P}A$ denote the probability of event A; indeed, Equation (1) may be approximated (10) as

\begin{equation*} \mathbb {P}\lbrace \mbox{$n$ RNA molecules are perfectly labelled}\rbrace \approx \exp \!\left( -\frac{n^2}{2N} \right), \end{equation*}

so that if a 0.9 probability of perfectly labelling *n* RNA molecules is desired, the number of pIDs required to achieve it is

\begin{equation*} N \approx -\frac{n^2}{2\log (0.9)}, \end{equation*}

i.e. it grows roughly quadratically in *n*.

**Figure 1.**
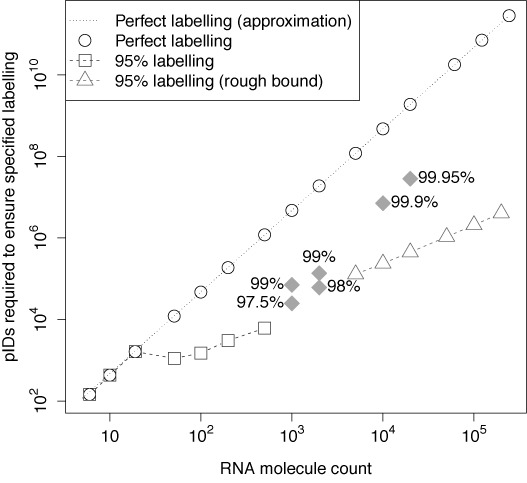
A log–log plot summarizing our labelling findings. The points marked by diamonds are the points we found by exact calculations for labelling better than 95% good.

Figure 1 illustrates the predicted number of pIDs that must be admitted by the pID design to ensure a 90% chance of achieving: perfect labelling, in which every RNA molecule receives its own pID; or 95%-good labelling. The shaded diamonds depict the numbers required for other levels of good labelling that we were able to compute. The above approximation to the number required for perfect labelling is drawn in a dotted line. In ‘deep sequencing’ applications of NGS that focus on the detection of rare variants, the number of RNA molecules is likely to exceed 10^3^; lower counts would diminish the need for NGS. At such numbers, one would require about 4.74 × 10^6^ pIDs to obtain a 90% chance of perfect labelling. A pID design would require at least 12 fully degenerate nucleotides to yield an adequate number of pIDs (Supplemental Materials, Figure S2). In contrast, a much lower number of pIDs (about 2.48 × 10^4^) would be required to have a 90% chance of attaining ‘97.5%-good’ (better than 95%-good) labelling of 10^3^ RNA molecules (Figure 1). Details of our calculations and tables containing detailed results are provided in Section S2.4 of the Supplemental Materials.

### Shallow read depths

To assess the suitability of the statistical model we devised for the read depths of observed RNA molecules, we applied it to the data from our serial dilution experiment targeting HIV reverse transcriptase. We found that the theoretical predictions made by the model appeared to at least follow the shape of the true observed data for the undiluted batches, although it performed progressively worse as the dilution factor increased; details of this validation may be found in Section S3.3 of the Supplementary Materials. We also applied the model to the HCV data; this, too, yielded mixed results (see Section S3.3 of the Supplementary Materials for details). Its performance on undiluted batches suggests that this model captures at least some of the true behaviour, so we proceed to use this model to make predictions on the number of RNA molecules that will be lost during error correction.

**Figure 2.**
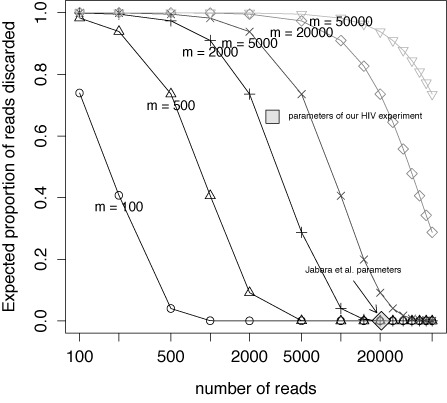
Theoretical expected proportion of reads discarded by rejecting singletons and doubletons using our model as described in Section S3.2 of the Supplemental Materials. The lines represent different values for the RNA molecule count *m*, and the *x*-axis represents the number of reads produced *N*. More RNA molecules mean more rejected reads for a fixed number of reads; more reads means fewer rejected reads for a fixed number of RNA molecules. The approximate parameters of (6) are marked with a shaded diamond; approximate parameters when using 12-way multiplexing of a 454 sequencing plate are marked with a shaded box.

Figure 2 shows the theoretical expected proportion of sequenced reads belonging to singleton or doubleton RNA molecules. Every singleton rejected translates to one read rejected, and every doubleton rejected translates to two reads rejected. It is apparent from this graph that even at moderate RNA molecule counts and numbers of reads, a substantial portion of sequenced reads belong to rejected RNA molecules. Large numbers of reads are necessary to avoid this. The approximate parameters used in (6) (∼2000 RNA molecules and ≥20 000 reads) are marked with a grey diamond; our model predicted that under their experimental conditions, almost none of their data would be rejected. (Supplementary Figure S3A of (6) indicates that roughly 30.5% of their observed pIDs were singletons and 7.5% were doubletons, indicating that across their total 72 162 reads, roughly 3% of their reads belonged to rejected RNA molecules. One possible cause for this discrepancy is sequencing error in the pID, causing two identical pIDs to be read as distinct.) In contrast, parameters approximating our current experimental results (∼2500 RNA molecules and 3000 reads) are marked with a grey box; in this regime more than half of the reads are expected to be discarded.

**Figure 3.**
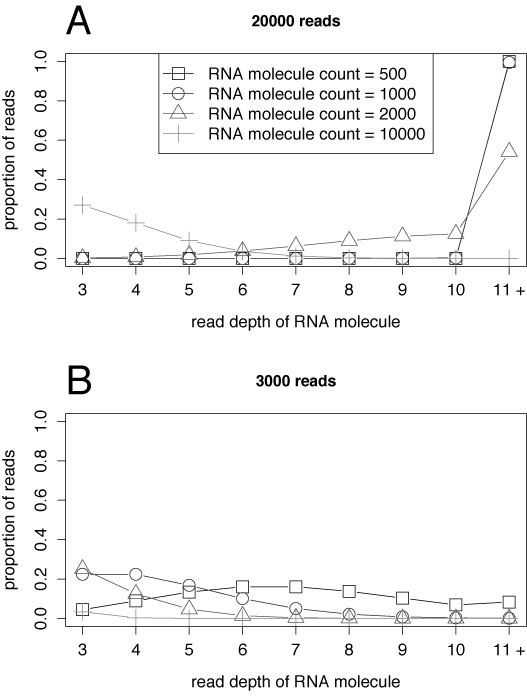
Expected proportion of reads belonging to *k*-RNAs for *k* = 3, 4, …, 10, 11 + for amplicon pools with the specified RNA molecule count where sequencing produced: (A) 20 000 total reads; and (B) 3000 total reads. Calculations were again done using our model as described in Supplementary Section S3.2. (A) reflects the conditions of (6); (B) roughly reflects the number of reads one might obtain from a single amplicon pool when using 12 sequencing barcodes to multiplex the sequencing plate on the 454 platform.

Figure 3 shows the expected proportion of reads belonging to *k*-RNAs coming from an amplicon pool producing 20 000 total reads, and the same for an amplicon pool producing 3000 total reads. The lines represent different RNA molecule counts *m* for the amplicon pool. As the RNA molecule count increases, reads tend to belong to pIDs with fewer representatives, eventually getting close to 0 for *k* > 1 while the number of singletons increases. From this we see that many more RNA molecules are expected to be retained after discarding singletons and doubletons when there are an ample number of sampled reads.

Table S11 of the Supplementary Materials shows the percentage of reads belonging to singleton and doubleton pIDs across all sequenced batches, and demonstrates how many reads may be rejected due to error correction. Note that these numbers are totalled across all sequenced batches, so they should not be directly compared to the results in Figure 2.

### Effects of pIDs on amplification and sequencing: microscopic

*Amplification and sequencing success*: Results from our generalized linear model analysis of amplification and sequencing success for the HIV experiment are summarized in Table S13 of the Supplementary Materials. It appears that, of the intrinsic factors we considered, none had significant effects. Success in amplifying and producing reads was much more affected by the extrinsic factors considered, indicating that bigger discrepancies come from differences in the RT, amplification and sequencing steps. This is not surprising in view of our analyses above; pID read depth is expected to increase as the number of reads from the batch increases and as the RNA molecule count decreases, and whether this happens or not is decided mostly at the level of the extrinsic factors (i.e. at the level of the batch and how it is processed).

In the HCV data we found that the base content of the pID had significant effects, with each of the C-, G- and T-content having significant associations with decreased success (C-content, *P* < 0.0001; G-content, *P* < 0.0001; T-content, *P* = 0.0061). See Table S16 of the Supplementary Materials for details.

*Indel probability*: The coefficients found in our logistic regression are shown in Table S14 of the Supplementary Materials. (Recall that our method for identifying indels was described in Section 2.)

First, we consider the intrinsic factors. We found that the homopolymer score (recall the definition from Supplementary Section S4.1) had a small but significant association with a higher rate of indel error (*P* < 0.0001): reads with higher homopolymer scores were more likely to produce indel errors, consistent with the 454 platform's known issues with homopolymer sequencing. The number of G's appearing in the pID also appeared to have a small but borderline significant effect, with error probability increasing as the number of G's increases (*P* = 0.052).

We next consider the extrinsic factors. The batch rVL had a significant but small effect, with higher batch rVLs significantly associated with a slightly higher error rate (*P* = 0.043). The pID design used also appeared to be significant, with the N9 and NBDHVBDHV designs being more indel-prone than the others (*P* < 0.0001). Only one sequencing barcode had a significant effect (*P* = 0.0013), with barcode F (sequence CGTGTCTCTA) associated with a slightly higher error rate over the baseline (barcode A, sequence ACGAGTGCGT). Certain plate regions also appeared to have significant effects. Plate region 2 was associated (*P* = 0.0034) with a higher probability of indel error than the baseline (plate region 1), while regions 3, 4 and 5 were all associated (*P* < 0.0001) with lower probabilities of indel errors than the baseline. Table S15 in the Supplemental Materials shows that region 2, in particular, exhibited a lower average read quality score than the other regions save for region 7, perhaps commensurate with its effect on error probability. Note, though, that these extrinsic factors may be confounded by uneven distribution of the other intrinsic factors.

### Effects of pIDs on amplification and sequencing: macroscopic

**Figure 4.**
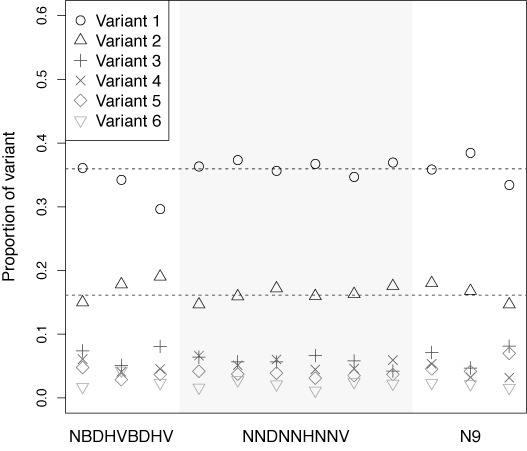
The prevalences of the top six variants observed in subject P1 across undiluted batches. The pID design used is denoted along the *x*-axis. For comparison, the overall prevalences of the top two variants are denoted with dashed lines.

Figure 4 shows the prevalences of the six variants we tracked for subject P1 across all undiluted batches. For reference, the prevalences of the top two variants (35% and 16%, respectively) are plotted as dashed lines. From this plot, no biases were immediately visually evident. Similarly, no biases were visually evident for subject P2 (not shown). Our multinomial logistic regression did indicate, however, some significant factors. The only significant design-related effect was on the most prevalent sequence, for which there was a significant (*P* = 0.029) increase in relative log odds from 0.072 when design NBDHVBDHV is used to 0.22 when design N9 was used. Dilution had a small but significant effect on the prevalences of two variants: for the second-most prevalent, a 10-fold decrease in concentration decreased the relative log odds by 0.095 (*P* = 0.041); and for the fifth-most prevalent, a 10-fold decrease in concentration decreased the relative log odds by 0.22 (*P* = 0.017). All coefficients of the regression may be found in Table S17 of the Supplementary Materials.

Using design N9 did disrupt the ordering of most to least probable, causing the fourth- and fifth-most prevalent variants to swap (except when the dilution was 100-fold). Note, though, that the effects of using design N9 on the fourth- and fifth-most prevalent variants were borderline significant at best. In all other settings, the ordering of variants by their probability did not change.

For P2, we performed the same fitting on the three most prevalent variants, noting that after removing batches producing too few reads the only batches that remained used either pID design NBDHVBDHV or NNNNNNNNN. The design used had no significant effect on any of the three variants (variant 1, *P* = 0.37; variant 2, *P* = 0.39; variant 3, *P* = 0.15), while dilution had a significant or borderline significant but small effect (variant 1, *P* = 0.026; variant 2, *P* = 0.051; variant 3, *P* = 0.015). None of these factors disrupted the ordering of the variants. See Table S18 of the Supplementary Materials for the regression coefficients.

**Figure 5.**
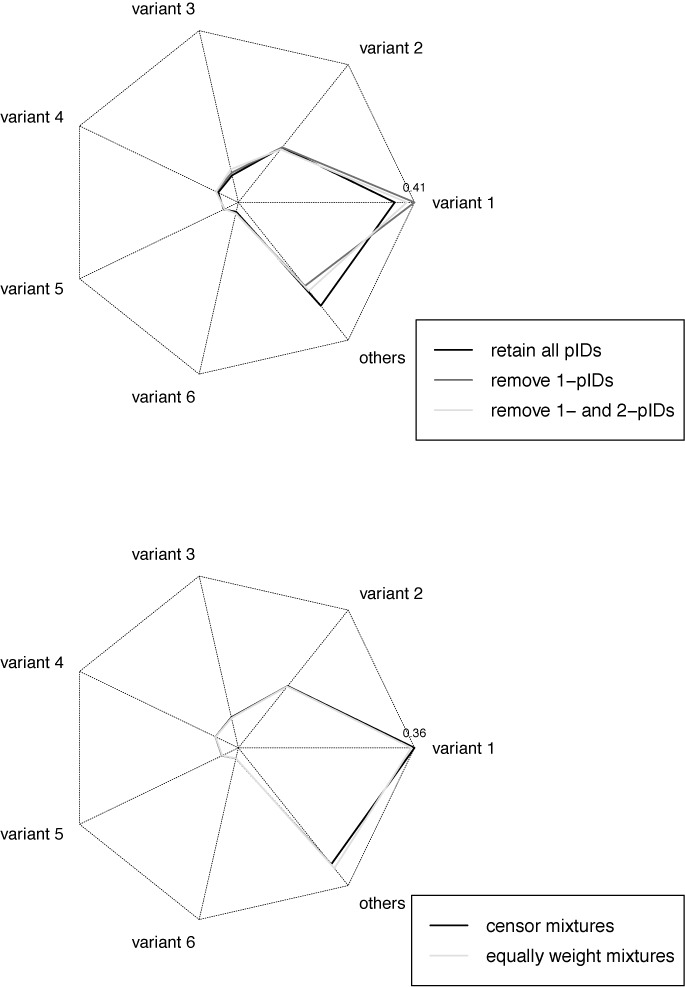
Star plots displaying the prevalences of the top six variants observed in subject P1 by keep scheme and by mixture resolution scheme, respectively. The distance along each line radiating from the centre represents the prevalence of each variant. In the former, a censoring mixture resolution scheme was used for all settings; in the latter, all pIDs were retained for all settings.

Next we consider the effect of error correction methods. Figure 5 shows the dependence of the prevalences of the top six variants observed in subject P1 on the keep scheme and the mixture resolution scheme employed. The keep scheme used had a notable effect on the prevalences of variants 1 and 3 especially, while the mixture resolution scheme appears to have had little effect. The multinomial logistic regressions supported this: changing the keep scheme from retaining all pIDs to removing singletons had a significant odds effect on the prevalences of several variants, including variants 1 and 3 (variant 1, *P* < 0.0001; variant 2, *P* = 0.0033; variant 3, *P* = 0.0026; variant 4, *P* = 0.0056; variant 5, *P* = 0.13; variant 6, *P* = 0.015), while changing it to removing singletons and doubletons had less significant effects, but still had a significant effect on variants 1 and 3 (variant 1, *P* = 0.0035; variant 2, *P* = 0.13; variant 3, *P* = 0.0053; variant 4, *P* = 0.055; variant 5, *P* = 0.27; variant 6, *P* = 0.068). Changing the keep scheme while holding fixed a censoring mixture resolution scheme had a large effect on the total number of RNA molecules observed: retaining all pIDs, 10 555 RNA molecules were identified across all P1 batches; rejecting singletons, the number was reduced to 1864; rejecting both singletons and doubletons further reduced the number to 950. Meanwhile, no significant effect was observed in changing the mixture resolution scheme from censoring to equal weighting (variant 1, *P* = 0.063; variant 2, *P* = 0.17; variant 3, *P* = 0.18; variant 4, *P* = 0.26; variant 5, *P* = 0.25; variant 6, *P* = 0.32). The mixture resolution scheme had a much smaller effect on the total number of RNA molecules observed: from a baseline of 10 555 when censoring to 11 084 when equally weighting mixtures (in both cases retaining all pIDs). See Supplementary Tables S19 and S20 for the regression coefficients. Also, see Section S5 of the Supplementary Materials along with the associated figures and tables for the analogous results for the top three variants of P2.

## DISCUSSION

Our sections on labelling and shallow read depths illustrate two issues that must be considered when using pIDs in NGS:

- having insufficiently many possible pIDs can lead to collisions in labelling that detract from the usefulness of the method; and
- a low read count relative to the RNA molecule count interferes with the ability to use pID-based consensus error correction.

In (6), the first issue was likely averted because they aimed to start their amplification reaction with 10 000 virus particles. If roughly 20% of these virus particles were eventually represented in their reads (based on the lab efficiency factors we estimated to describe the proportion of RNA molecules that successfully amplify—see Section S3.3 of the Supplemental Materials), then the resulting RNA molecule count was likely on the order of 2000, which Figure S2 in the Supplementary Materials shows can be well covered by the 65 536 possible pIDs admitted by their pID design (N8). Our calculations indicate that care must be taken in the pID design to admit enough possible pIDs. For example, in Section S3.3 of the Supplemental Materials, we saw that labelling collisions were possibly a factor in observing fewer singletons and doubletons than theoretically expected because multiple distinct RNA molecules might have been attached to the same pID. If this happens, then the ability to effectively track distinct RNA molecules may be compromised. The problem will also occur when using samples with very high viral loads. Sample rVLs may exceed 10^6^ HIV RNA copies/ml; the procedure followed in this study might yield amplicon pools with a RNA molecule count of upwards of 30 000. At that point, the length of the pID design required to admit a sufficiently high number of pIDs to avoid collisions may become prohibitive. One possible workaround with such samples is to dilute them down so that the RNA molecule count does not exceed the design's ‘capacity’ (this is done in (6)); if one needs to observe more RNA molecules than this diluted sample produces after sequencing, then one can sequence several replicate batches.

Jabara *et al.* avoided the second problem because upwards of 20 000 reads were generated for each amplicon pool considered (6). However, Roche/454 sequencing runs often use sequencing tags to allow several amplicon pools to be run on the same plate (11–13), as our procedure did, with the tradeoff that fewer reads can be generated per amplicon pool. Optimistically, when 12-way multiplexing is used as in our HIV experimental procedure, ∼2000–3000 reads can be generated per amplicon pool; Figures S3 and S4 of the Supplementary Materials show that the number can frequently be significantly less than this. Under such conditions, if the RNA molecule counts are high (for example, if the source sample has a high viral load), discarding singletons and doubletons may lead to the rejection of the bulk of the reads produced. This also affected our HCV data, where despite higher numbers of reads per batch, rejecting singletons and doubletons still discarded a large proportion of data (see Supplementary Figure S6), possibly due to unexpectedly high RNA molecule counts. This problem may be mitigated by the use of a different NGS platform that generates much higher read counts than the 454, such as the Illumina MiSeq.

Our results in ‘Effects of pIDs on Amplification and Sequencing: Microscopic’ indicate that the occurrence of homopolymers in pIDs did not have much bearing on their success in achieving high read depths in amplification or sequencing; such success appeared to be largely influenced by extrinsic factors, such as the RT and amplification chemistry and the sequencing. (This is another place where an NGS platform generating higher read counts than the 454 should help.) Nucleotide content did not have a significant association with sequencing success in our HIV data, but did in our HCV data. We did find that the occurrence of homopolymers in pIDs had a small but significant effect on indel probability, which is likely at least partly due to the known problems of the 454 platform in handling homopolymers; we also found the G content of the pID to have a small but significant effect. This indel probability was also affected by several extrinsic factors. Note that our method of detecting indel errors did not attempt to identify pIDs with more than one indel error, or with indel errors of apparent length longer than one. Such pIDs would be more likely to resolve to multiple proper pIDs, leading us to have less confidence in our resolution. Indeed, these corrections already make an arbitrary decision about which proper length pID to resolve to when there are multiple possibilities, which can cause the wrong pID to be recorded as having or not having an indel error. Moreover, our method removed pIDs which might otherwise be resolvable to a ‘proper’ length 9 pID but for substitution errors. It also ignores the possibility of errors being introduced before the sequencing stage, for example, in reverse transcription or in amplification. Another limitation is that pIDs of length 8 or 10 that result from an indel error in sequencing a length 9 pID cannot be identified if the proper length 9 pID is not sequenced. Particularly in the cases with high RNA molecule counts, this could easily be a factor because there is a good chance that the proper pID does not produce any other reads. Also, it is worth noting that using pIDs does not address indel errors that are systematically mishandled by the data processing pipeline. In the future it may be revealing to consider the effects of other intrinsic factors, such as secondary structure and the presence of hairpins, or primer dimers, as well as other extrinsic factors.

We also find in ‘Effects of pIDs on Amplification and Sequencing: Macroscopic’ that the choice of pID design did not appear to greatly bias to the data, nor did the dilution of the batch. Our visual interpretation was supported by a multinomial logistic regression on these factors. The error correction scheme did, however, introduce slight but significant bias to the data, with different schemes for retaining pIDs based on read depth having significant effects on the prevalences of top variants observed for both HIV subjects. Changing the keep scheme also greatly reduced the number of RNA molecules observed, which may partially explain the effects on sequence diversity. Meanwhile, changing the mixture resolution scheme appeared to have only insignificant effects for subject P1, and had a small but significant effect for the top variant observed in subject P2. Note that the variants we considered were identified after trimming our reads to a 100 bp region using automated pairwise alignment. This was necessary to avoid regions of RT which are highly susceptible to sequencing error, but may have misidentified the desired portion of the sequence in some reads.

We believe that using pIDs can be of great value in using NGS. The problems they solve—error correction of noisy NGS data and quantification of the RNA molecules sequenced—are both important technical factors that must be addressed if we want to collect the highest quality data possible. Our findings are reminders that, as with any new technology, care must be taken in the implementation of pIDs. We have focused primarily on purely theoretical limitations of the method in our sections on labelling and shallow read depths; these are hard limitations imposed by the randomness in the procedure that cannot be worked around, and so we must be aware of them. Other practical problems and deviations from the assumptions we made are bound to occur as sequencing technology moves forward, and those issues must be found in turn; we are only scratching the surface in our examination of the effects of pIDs on sequencing and analysis.

## SUPPLEMENTARY DATA

Supplementary Data are available at NAR Online.

Supplementary Data
