# Supplementary material for: Theoretical and experimental assessment of degenerate primer tagging in ultra-deep applications of next-generation sequencing
Source: Nucleic Acids Res. 2014 May 7;42(12):e98. doi: 10.1093/nar/gku355 (PMC4081055; doi:10.1093/nar/gku355)
Supplement: Supplementary Data [file supp_42_12_e98__index.html]

Theoretical and experimental assessment of degenerate primer tagging in ultra-deep applications of next-generation sequencing — Theoretical and experimental assessment of degenerate primer tagging in ultra-deep applications of next-generation sequencing — Supplementary Data 

# Theoretical and experimental assessment of degenerate primer tagging in ultra-deep applications of next-generation sequencing

## Supplementary Data

**Files in this Data Supplement:**

- Supplementary Data
