# Supplementary material for: Theoretical and experimental assessment of degenerate primer tagging in ultra-deep applications of next-generation sequencing
Source: Nucleic Acids Res. 2014 May 7;42(12):e98. doi: 10.1093/nar/gku355 (PMC4081055; doi:10.1093/nar/gku355)
Supplement: Supplementary Data [file supp_gku355_nar-01032-met-k-2013-File002.pdf]

Theoretical and experimental assessment of degenerate primer tagging in ultra-deep applications of next-generation sequencing: supplementary materials

Richard H. Liang[1], Theresa Mo[1], Winnie Dong[1], Guinevere Q. Lee[1], Luke C. Swenson[1], Rosemary M. McCloskey[1], Conan K. Woods[1], Chanson J. Brumme[1], Cynthia K.Y. Ho[3], Janke Schinkel[3], Jeffrey B. Joy[1], P. Richard Harrigan[1,2], and Art F.Y. Poon[1,2]

[1]BC Centre for Excellence in HIV/AIDS, Vancouver, BC, Canada
[2]Department of Medicine, University of British Columbia, Vancouver, BC, Canada
[3]Section of Clinical Virology, Department of Medical Microbiology, Academic Medical Center, Amsterdam, The Netherlands

April 3, 2014

S1 Wet bench methodology

S1.1 HIV experiment

RNA extraction was performed using a NucliSENS easyMAG (bioMerieux) and reverse transcription was performed using Expand Reverse Transcriptase (Roche). It is during this step that the primer ID tags were attached, along with sequencing barcodes. Following this, a primer removal step using AMPure magnetic beads (Beckman Coulter) was performed. Two rounds of PCR, using the Expand High Fidelity PCR system (Roche), were then applied to amplify the resulting cleaned cDNA. Fusion primers were attached at the reverse transcriptase step and during the second round of PCR on the 3' and 5' ends of the strand, respectively, to enable the strands to fuse to the emulsion PCR beads used in 454-based sequencing. After amplification, all PCR products were quantified using a Quant-iT Picogreen dsDNA Assay Kit (Invitrogen) and a DTX 880 Multimode Detector (Beckman Coulter), and pooled to be equimolar. The resulting product was then attached to sequencing beads by emulsion PCR (Lib-A, Roche). Our primers were made by Integrated DNA Technologies, with HPLC purification; primer designs are outlined in table S1. Experimental conditions of the reverse transcription and PCR steps, as well as thermal cycler protocols and the cDNA cleanup procedure, are given in tables S2 and S3.

S1.2 HCV experiment

Coverage: We calculated that the minimum required coverage for the detection of a 0.1% minor variant with 95% confidence, taking into account the reads lost due to QC, was 15 000 reads per batch. This estimate was based on a 50% data loss observed in a previous NGS experiment and that the number of unique RNA molecules sequenced was 40% of the total number of reads [1]. For the final plate setup the calculated coverage was 22 500 reads per batch.

RNA extraction: RNA was extracted with the QIAamp viral RNA mini kit (Qiagen) from serum samples with viral load $\geq \log 4$. Viral loads were measured with the CAP/CTM v2.0 in IU/ml and were converted to copies/µl by multiplication with 5.2×10^{-3}. To obtain 10 000 copies, we eluted an amount of serum that would contain 12 000 copies in 12 µl elution buffer and extracted 10 µl.

GS-FLX Titanium primer A

`5' CGTATCGCCTCCCTCGCGCCA 3'`

GS-FLX Titanium primer B

`5' CTATGCGCCTTGCCAGCCCGC 3'`

Sequencing barcodes

(A)	ACGAGTGCGT
(B)	ACGCTCGACA
(C)	AGACGCACTC
(D)	AGCACTGTAG
(E)	ATCAGACACG
(F)	CGTGTCTCTA
(G)	CTCGCGTGTC
(H)	TAGTATCAGC
(I)	TCTCTATGCG
(J)	TGATACGTCT
(K)	TACTGAGCTA
(L)	ATATCGCGAG

Reverse primer for cDNA synthesis

5' [Titanium primer B] - [Library tag] - [Barcode] - [Primer ID] - TTTGYTCTATGCTGCCCTAT 3'

example: 5' CTATGCGCCTTGCCAGCCCGC TCAG ACGAGTGCGT NNDNNHNNV TTTGYTCTATGCTGCCCTAT 3'

First-round PCR forward primer

5' AATTGGGCCTGAAAATCC 3'

First-round PCR reverse primer

5' [Titanium primer B] 3'

actual: 5' CTATGCGCCTTGCCAGCCCGC 3'

Second-round (nested) PCR forward primer

5' [Titanium primer A] - [Library tag] - [Barcode] - GGAAGTTCAATTAGGAATACC 3'

example: 5' CGTATCGCCTCCCTCGCGCCA TCAG ACGAGTGCGT GGAAGTTCAATTAGGAATACC 3'

Second-round (nested) PCR reverse primer

5' [Titanium primer B] - [Library tag] - [Barcode] 3'

example: 5' CTATGCGCCTTGCCAGCCCGC TCAG ACGAGTGCGT 3'

Table S1: Primer designs used in our experiment. The sequencing barcodes used are also listed. Barcode A and primer ID design NNDNNHNNV are used in all of the examples. The library tag TCAG is used in 454 sequencing and must be attached after the fusion primer to denote that the bead belongs to the library and is not a control bead.

Reverse transcription mix

	amount (µl)
DEPC treated water	1.5
Expand High Fidelity Buffer 10X (15 mM $MgCl_2$)	1
$MgCl_2$, 25 mM	1.4
dNTP, 25 mM	0.4
Protector RNase inhibitor, 40 U/µl	0.2
Expand Reverse Transcriptase, 50 U/µl	0.2
cDNA synthesis primer, 25 mM	0.3
	5
extract	5
total	10

Thermal cycler protocol
45′ for 43 °C

cDNA cleanup (primer removal) procedure:

- add 10 µl DEPC treated water to the product
- mix with 40 µl AMPure magnetic bead, incubate 5 minutes at room temperature
- 4 washes with 200 µl 70% ETOH
- air dry, and suspend beads in 37 µl DEPC treated water

Table S2: Mix, thermal cycler protocol, and cDNA cleanup procedure used for reverse transcription. The Expand Reverse Transcriptase, Expand High Fidelity Buffer, $MgCl_2$ stock solution, Protector RNase Inhibitor, and dNTP were all from Roche.

1st round PCR mix

	amount (µl)
50% sucrose, 0.04% bromophenol blue solution	5
Expand High Fidelity Buffer 10X (15 mM MgCl2)	5
MgCl2, 25 mM	2
dNTP, 25 mM	0.4
Expand High Fidelity PCR Enzyme Mix, 3.5 U/µl	0.72
First-round PCR forward primer, 25 mM	0.38
First-round PCR reverse primer, 25 mM	0.38
	13.87
purified cDNA beads	≈ 36
total	50

2nd round nested PCR mix

	amount (µl)
DEPC treated water	13.45
60% sucrose, 0.08% cresol red solution	2
Expand High Fidelity Buffer 10X (15 mM MgCl2)	2
MgCl2, 25 mM	0.8
dNTP, 25 mM	0.16
Expand High Fidelity PCR Enzyme Mix, 3.5 U/µl	0.29
Second-round PCR forward primer, 25 mM	0.15
Second-round PCR reverse primer, 25 mM	0.15
	19
1st-round PCR product	1
total	20

Thermal cycler protocol
2′ @ 94 °C
10 × (15″ @ 94 °C, 30″ @ 55 °C, 1′ @ 72 °C)
25 × (15″ @ 94 °C, 30″ @ 55 °C, 1′* @ 72 °C) * increase 5″ per cycle
7′ @ 72 °C

Table S3: Mixes used for both rounds of PCR, as well as the thermal cycler protocol used for both rounds. The Expand High Fidelity Buffer, Expand High Fidelity PCR Enzyme Mix, MgCl2 stock solution, and dNTPs were all from Roche. Note that the 1st-round reaction tube was placed on a magnetic bar before transferring product into the 2nd-round PCR mix.

RT reaction: The RT reaction was performed with the antisense primer DM100 (H77 8616-8638) [2] which was ligated to the pID (N9), sequencing barcode (10nt), and the 454 adaptor A sequence (Lib-L, Roche). Superscript III (Invitrogen) was used according to the manufacturer's protocol with modifications on the incubation temperature (65 °C), with a primer concentration of 50 µM per reaction, and omitting the RnaseH step. The input of the RT reaction was 10 µl elution containing 10 000 RNA templates and the end volume was 20 µl.

PCR reaction: To fully utilize the RT products (20 µl) four PCRs, each starting with 5 µl of RT product, were performed per sample using the Transcriptor High Fidelity cDNA synthesis kit (Roche). Amplicons were pooled afterwards. The DM101 (H77 8250-8275) primer [2], ligated to the 454 adaptor B sequence (Lib-L, Roche), was used as the sense primer and the 454 adaptor A sequence as the antisense primer. The reaction mixes were prepared according to the manufacturers protocol with a modified primer concentration resulting in 0.1 µl primer per reaction and an end volume of 25 µl. PCR cycle conditions were: $5'$ 95 °C; then 45 cycles of 95 °C for $30''$, 55 °C for $30''$, and 72 °C for $90''$; finally, a $10'$ extension step at 72 °C. Amplicon purification was performed twice with AMPure XP beads (Beckman Coulter) and the product size was quantified using the Agilent DNA kit (Agilent Technologies). DNA concentration was measured with the Qubit dsDNA HS assay kit (Life technologies). The primers were produced by Biolegio, with PAGE purification.

454 sequencing: Amplicon pools were diluted to 1.108 molecule/µl and pooled. The pooled amplicon library was diluted further to 1.106 molecule/µl and sequenced on the 454 GS FLX platform with the XLR70 Titanium sequencing chemistry and the "one-way read" Lib-L method according to the manufacurers protocol (Roche).

S2 Labelling

S2.1 Perfect labelling

Table S5 shows the number of pIDs required to achieve a high probability (0.9) of perfect labelling for selected RNA molecule count values. Certain values in the table – 10, 100, 1000, and 10 000 – were chosen as reasonable RNA molecule counts for the amplicon pools that one might put into a Roche/454 sequencing run. The values 51 and 199 are given because they represent the interval of likely *true* RNA molecule counts for a *reported* RNA molecule count of 100, taking into account the error inherent in a viral load assay: such assays are only considered accurate to within 0.3 $\log_{10}$ copies/ml. Similarly for values such as 502, 1995, etc.

The value 243 954 represents the largest possible RNA molecule count that we could achieve high probability of perfect labelling for using a design consisting entirely of Bs, Ds, Hs, and Vs (so that there are three possibilities for each position) of length 24, three times a "typical" design length. (Such a design guarantees that any homopolymer in the pID is of length at most 3.) This gives us a total of $3^{24} = 282\,429\,536\,481$ possible primer ID tags. Therefore 122 267 is the largest reported RNA molecule count that can be confidently handled using this design, as a reported RNA molecule count of 122 267 may well result in as large of a true RNA molecule count as 243 954 due to the aforementioned uncertainty in viral load estimation. (This would also require all of the RNA molecules to successfully reverse transcribe and amplify.) Considering that HIV samples may have plasma viral loads (pVL) in the millions or even higher, and that a typical amplicon pool produced via the method described in section 2 might capture roughly 4% of the virions in the sample, such an RNA molecule count is far from extreme.

S2.2 Exact bounds

Recall the definition of k-tags from section 2. Suppose that there are n RNA molecules and N unique pIDs, all equally likely to attach to any RNA molecule. We may think of each pID as being in infinite supply, so that the assignment of pIDs to RNA molecules is done as sampling with replacement. Let us count the number of assignments of RNA molecules and pIDs where the number of k-tags, for $k = 1, \ldots, n$, is t_k. Note

Sequencing barcodes					
(1)	ACGAGTGCGT	(16)	TCACGTACTA	(30)	AGACTATACT
(2)	ACGCTCGACA	(18)	TCTACGTAGC	(31)	AGCGTCGTCT
(3)	AGACGCACTC	(19)	TGTACTACTC	(33)	ATAGAGTACT
(5)	ATCAGACACG	(20)	ACGACTACAG	(34)	CACGCTACGT
(7)	CGTGTCTCTA	(22)	TACGAGTATG	(35)	CAGTAGACGT
(10)	TCTCTATGCG	(23)	TACTCTCGTG	(36)	CGACGTGACT
(11)	TGATACGTCT	(24)	TAGAGACGAG	(37)	TACACACACT
(120)	AGTGAGCTCG	(25)	TCGTCGCTCG	(38)	TACACGTGAT
(13)	CATAGTAGTG	(26)	ACATACGCGT	(39)	TACAGATCGT
(14)	CGAGAGATAC	(28)	ACTACTATGT	(40)	TACGCTGTCT
(15)	ATACGACGTA	(29)	ACTGTACAGT		

Lib-L adaptor sequence A
5' CCATCTCATCCCTGCGTGTCTCCGAC 3'
Lib-L adaptor sequence B
5' CCTATCCCCTGTGTGCCTTGGCAGTC 3'
DM101 [2]
5' TTCTCRTATGAYACCCGCTGYTTTGA 3'
DM100 [2]
5' TACCTVGTCATAGCCTCCGTGAA 3'
Reverse primer for cDNA synthesis
5' [Lib-L adaptor A] - [Library tag] - [Barcode] - [Primer ID] - [DM100] 3'
example: 5' CCATCTCATCCCTGCGTGTCTCCGAC TCAG ACGAGTGCGT NNNNNNNNN TACCTVGTCATAGCCTCCGTGAA 3'
Forward (sense) PCR primer
5' [Lib-L adaptor B] - [Library tag] - [DM101] 3'
example: 5' CCTATCCCCTGTGTGCCTTGGCAGTC TCAG TTCTCRTATGAYACCCGCTGYTTTGA 3'
Reverse (antisense) PCR primer
5' [Lib-L adaptor A] - [Library tag] 3'
example: 5' CCATCTCATCCCTGCGTGTCTCCGAC TCAG 3'

Table S4: Primers and sequencing barcodes used in our HCV experiment. Sequencing barcode 1 is used in the example for the cDNA synthesis primer. The library tag is used in 454 sequencing as for the primers in table S1, for the same purpose. The sequencing barcode sequences are taken from [3].

RNA molecule count	pIDs required	Minimum design length
10 (6; 19)	431 (145; 1630)	6
100 (51; 199)	47 015 (12 119; 1.871×10^5)	9
1000 (502; 1995)	4.741×10^6 (1.194×10^6; 1.888×10^7)	13
10 000 (5012; 19 952)	4.7452×10^8 (1.192×10^8; 1.889×10^9)	16
122 267 (61 279; 243 954)	7.094×10^{10} (1.782×10^{10}; 2.824×10^{11})	20

Table S5: Theoretically obtained number of pIDs required to ensure 0.9 probability of perfect labelling for selected values of RNA molecule count. In the first column, the values in parentheses represent the interval of likely true RNA molecule counts for the reported RNA molecule count outside parentheses. The corresponding values of pIDs required are also in parentheses in the second column, and the minimum design length required to assure enough possible pIDs (for the maximum value in parentheses) is in the third column.

that we are subject to the constraint that

$$\sum_{k=1}^{n} kt_k = n.$$

We number the RNA molecules $1, \ldots, n$ and number the pIDs $1, \ldots, N$. The number of such assignments can be counted as follows:

- Pick t_1 RNA molecules to be assigned to 1-tags, $2t_2$ RNA molecules from those remaining to be assigned to 2-tags, and so on and so forth. The number of ways to do this is given by the multinomial coefficient

$$\binom{n}{n, \ldots, n, n-1, \ldots, n-1, \ldots, 2, \ldots, 2, 1, \ldots, 1}$$

  where the number of times k occurs in the lower part of the multinomial coefficient is t_k. (Note that in the above, n could only appear once in the lower part of the multinomial coefficient.) Here, we are picking the RNA molecules that get assigned to k-tags in turn, so that we have a first k-subset of RNA molecules that will receive the smallest-numbered of the k-tags, a second k-subset of RNA molecules that will receive the next smallest-numbered of the k-tags, and so on up to the t_kth such subset.

- Pick t_1 pIDs to be the t_1 1-tags, t_2 pIDs to be the t_2 2-tags, and so on. The number of ways to do this is

$$\binom{N}{t_1, t_2, \ldots, t_n, N - \sum_{j=1}^{n} t_j},$$

  where the last term on the bottom represents all of the unchosen pIDs.

We multiply these two factors together to get the number of desired assignments. The probability of any single assignment is simply $1/N^n$: under this model all assignments are equally likely, and there are N^n of them (assign a pID to each RNA molecule in turn with no restrictions). Therefore, letting $\mathbb{P}$ denote probability,

$$\begin{aligned}
&\mathbb{P}\{(\text{number of } k\text{-tags})_k = (t_1, t_2, \ldots, t_n)\} \\
&= \binom{n}{n, \ldots, n, n-1, \ldots, n-1, \ldots, 2, \ldots, 2, 1, \ldots, 1} \\
&\quad \times \binom{N}{t_1, t_2, \ldots, t_n, N - \sum_{j=1}^{n} t_j} \frac{1}{N^n},
\end{aligned} \tag{S1}$$

where as above the number of times k appears in the lower part of the multinomial coefficient is t_k. We call such a set of assignments, where the number of k-tags is t_k for each k, a *k-tag configuration*, or simply a *configuration*.

Recalling the definition of 95% good labelling from section 2, we used formula (S1) to compute the probability of 95% good labelling for a given N and n. To do this, we first enumerated all of the possible configurations that can lead to good labelling. For example, if there are 100 RNA molecules, some of the configurations that constitute 95% good labelling are:

- 100 1-tags;
- 98 1-tags and a single 2-tag;
- 97 1-tags and a single 3-tag;
- 96 1-tags and two 2-tags;
- 96 1-tags and a single 4-tag;
- 95 1-tags, one 2-tag, and one 3-tag;

- 95 1-tags and a single 5-tag;

and so on.

Once the configurations were enumerated, we applied equation (S1) to compute their probabilities. Specifically, for a given number of RNA molecules, our goal was to have a 0.9 probability (90%) of achieving 95% good labelling, and we were looking for the number of unique pIDs required to do so. That is, if $p(N)$ is the probability of achieving 95% good labelling when N unique pIDs are employed, our goal was to find a value N^* such that $p(N^*) \geq 0.9$ and $p(N^* - 1) < 0.9$. The algorithm we used is as follows.

- We start with an initial guess at an interval $(N_{\min}, N_{\max}]$ that contains N^*.
- If $p(N_{\min}) \geq 0.9$ then we recurse on the interval $(N_{\min}/2, N_{\min}]$.
- Otherwise, if $p(N_{\max}) < 0.9$ then we recurse on the interval $(N_{\max}, 2N_{\max}]$.
- Once an interval is found so that $p(N_{\min}) < 0.9 \leq p(N_{\max})$, we then employ a bisection search heuristic, recursively cutting the interval in half until we arrive at a solution N^*.

It can be shown that p is strictly increasing for all N, and that $p(N) \uparrow 1$ ($p(N)$ is bounded below by the probability of perfect labelling, which does increase to 1). Therefore, a solution N^* exists, and our algorithm finds the optimal value.

S2.3 Inexact bounds

As mentioned in section 2, the above method is too computationally expensive to perform for large RNA molecule counts, so we computed approximations in those cases: here, we describe the approximation method. Some mathematical notation that will be used frequently in the following: if we have a description of an event in set notation like $\{(\text{description})\}$, then we will write the indicator function of that event as simply $\mathbf{1}\{(\text{description})\}$. We will write $\mathbb{E}X$ and $\text{var}X$ for the expected value and variance of a random variable X, respectively.. Let T_1 be the number of RNA molecules that have unique pIDs; we can represent it as

$$T_1 = \sum_{i=1}^{n} \mathbf{1}\{\text{RNA molecule } i \text{ receives a unique pID}\}. \tag{S2}$$

Let $\mu = \mathbb{E}[T_1]$ and let $\sigma^2 = \text{var}(T_1)$. Chebyshev's inequality tells us that

$$\mathbb{P}\Big\{-\sqrt{10}\sigma \leq T_1 - \mu \leq \sqrt{10}\sigma\Big\} \geq 0.9.$$

A fortiori, then,

$$\mathbb{P}\Big\{T_1 \geq \mu - \sqrt{10}\sigma\Big\} \geq 0.9.$$

If we fix the number of RNA molecules n, then μ and σ are both functions of N, with $\mu \uparrow n$ and $\sigma \to 0$ as $N \uparrow \infty$. If we can find N such that $\mu - \sqrt{10}\sigma \geq 0.95n$, then the above tells us that

$$\mathbb{P}\{T_1 \geq 0.95n\} \geq 0.9. \tag{S3}$$

If $T_1 \geq 0.95n$, that is, if the number of RNA molecules that have unique pIDs is at least 95% of all RNA molecules, *then we certainly have* 95% *good labelling.* Therefore such an N gives a rough bound on the number of pIDs needed to assure 0.9 probability of 95% good labelling, the number we seek. That is, if we choose a pID design providing at least this many possible pIDs, then we will have the 0.9 probability of 95% good labelling that we have been aiming for.

The calculations of $\mathbb{E}[T_1]$ and $\text{var}(T_1)$ are a matter of combinatorics. We start with $\mathbb{E}[T_1]$. Consider one RNA molecule; the probability that this one RNA molecule is assigned to a unique primer ID is $((N -$

$1)/N)^{n-1}$, as the first RNA molecule can be assigned to any primer ID, and all $n-1$ other RNA molecules must be assigned to any other primer ID. Therefore

$$\begin{aligned}\mathbb{E}[T_1] &= \sum_{i=1}^{n} \mathbb{P}\{\text{RNA molecule } i \text{ receives a unique pID}\} \\ &= n\left(\frac{N-1}{N}\right)^{(n-1)}. \end{aligned} \tag{S4}$$

We compute $\mathrm{var}(T_1) = \mathbb{E}\left[T_1^2\right] - \mathbb{E}[T_1]^2$ by first computing $\mathbb{E}\left[T_1^2\right]$. Let $\mathbf{1}_i$ denote the indicator of the event that RNA molecule i receives a unique pID. First, note that

$$\begin{aligned}T_1^2 &= \sum_{i=1}^{n}\sum_{j=1}^{n} \mathbf{1}_i \mathbf{1}_j \\ &= 2 \sum_{1 \le i < j \le n} \mathbf{1}\{\text{RNA molecules } i \text{ and } j \text{ both receive unique pIDs}\} + \sum_{i=1}^{n} \mathbf{1}_i.\end{aligned}$$

The second sum in the last line is simply T_1. The events represented by the indicators in the first sum of the last line have probabilities

$$\mathbb{P}\{\text{RNA molecules } i \text{ and } j \text{ both receive unique pIDs}\} = \frac{N-1}{N}\left(\frac{N-2}{N}\right)^{(n-2)}.$$

To see this, note that RNA molecule i may be assigned to any pID, while RNA molecule j may be assigned to any other pID, which happens with probability $(N-1)/N$. Every other RNA molecule must then be assigned to a pID which is not either of the pIDs assigned to RNA molecules i and j; each RNA molecule has probability $(N-2)/N$ of this occurring. Putting this into the previous calculation and using equation (S4) gives that

$$\begin{aligned}\mathrm{var}(T_1) = n(n-1)\frac{N-1}{N}\left(\frac{N-2}{N}\right)^{(n-2)} &+ n\left(\frac{N-1}{N}\right)^{(n-1)} \\ - n^2\left(\frac{N-1}{N}\right)^{(2n-2)}&. \end{aligned} \tag{S5}$$

We utilized a similar search heuristic to the one used in finding exact bounds to find an N that satisfied equation (S3). Here, we do not have a guarantee that $\mu - \sqrt{10}\sigma$ is strictly increasing in N, but we can find using calculus that it reaches an increasing regime when N is large. In fact we do not even require this, but only require the fact that $\mu - \sqrt{10}\sigma \longrightarrow n$ to guarantee that our search finds a solution (that may not be the optimal one). Because we know that $\mu - \sqrt{10}\sigma$ eventually reaches a strictly increasing regime, and because we expect that our solutions are likely in this regime, we believe that our solution is likely close to the optimal.

S2.4 Results: supplementary details

We attempted to carry out the search for the number of pIDs needed for 95% good labelling for the same RNA molecule counts considered in Table S5, but encountered computational hurdles: as the RNA molecule count increases, so too does the number of configurations we need to consider. For reference, even enumerating the configurations required to compute this number for 1000 RNA molecules exhausted the system memory (16GB) of the computer we ran the code on, while the number of configurations required to compute this number for 502 RNA molecules only took roughly 100MB of memory. Our results are in table S6; these results were the ones we were able to obtain in roughly two weeks of computing time on a 2.8 GHz quad-core

RNA molecule count	pIDs required	Minimum design length
6	145	4
10	431	5
19	1630	6
51	1113	6
100	1499	6
199	3042	6
502	6121	7

Table S6: Theoretically-obtained number of pIDs required to ensure 0.9 probability of 95% good labelling for selected values of RNA molecule count. For reference, the shortest primer ID design that provides this number of pIDs is shown in the third column (based on a design Nm, which provides the maximum number of pIDs for a given length). Note that more pIDs are required for 19 RNA molecules than for 100 RNA molecules; this is because 95% good labelling of 19 RNA molecules is the same as perfect labelling, while with 100 RNA molecules we have some margin for error. Compare with table S5.

RNA molecule count	Quality of labelling	Possible pIDs required	Minimum design length
1000	97.5% (975 tags)	24 822	8
1000	99% (990 tags)	70 531	9
1995	98.0% (1956 tags)	60 938	8
1995	99.0% (1976 tags)	135 860	9
10 000	99.9% (9990 tags)	7 114 876	12
19 952	99.95% (19 942 tags)	28 336 710	13

Table S7: Number of pIDs required to ensure 0.9 probability of the specified number of distinct pIDs being represented in an amplicon pool with the given RNA molecule count; minimum required design length is in the fourth column. *Note:* for 1995 RNA molecules and 1956 pIDs represented, we had to terminate the calculation before it found the best possible value, and 60 938 is only an upper bound; however, the final result would have been no smaller than 60 935. All other calculations ran to completion.

Xeon processor. (Note that each run did not use all four cores; each physical core is treated as two logical cores, and each job only used one of eight logical cores. Jobs were run simultaneously where possible given the memory constraints.) Of note in this table is the fact that fewer pIDs are needed to provide 95% good labelling for 51 RNA molecules than for 19 RNA molecules. This is due to a rounding issue: 95% good labelling of 19 RNA molecules is in fact perfect labelling, because if only 18 distinct pIDs are represented, for example, then we have only achieved 94.7% good labelling. With 51 RNA molecules we have some margin for error in how pIDs can be assigned to RNA molecules.

For larger RNA molecule counts, we attempted to pare down the computation time by considering fewer configurations. To do this, we considered events with *better* labelling than 95% good labelling; i.e. we considered the probability of getting cleaner data than we would have with 95% good labelling. This is more conservative than we would like to be, but we proceeded with the hope that the number of primer IDs required would still be vastly reduced from those needed to ensure high probability of perfect labelling. We were still unable to compute values for the largest RNA molecule counts considered in table S5, but were nonetheless able to glean some useful information: see table S7.

Comparing tables S6 and S7 to table S5 shows, encouragingly, that if we accept that perfect labelling is impractical, it takes significantly fewer pIDs – and therefore a shorter and/or simpler design – to ensure 95% good labelling or better. For example, to ensure 99.9% good labelling on an amplicon pool with an RNA molecule count of 10 000, we only need to make sure that there are 7 114 876 primer IDs available; to get perfect labelling requires nearly 67 times more. For reference, the former requires a primer ID of length at least 12 (a design including 11 Ns and 1 R, for example, would yield $4^{11} \times 2 = 8\,388\,608$ primer IDs),

RNA molecule count	pIDs required	Minimum design length	Quality of labelling *in silico*
1000	36 196	8	98.19% (982 pIDs)
1995	60 407	8	97.99% (1955 pIDs)
5012	129 057	9	97.85% (4904 pIDs)
10 000	237 390	9	97.75% (9775 pIDs)
19 952	447 132	10	97.68% (19489 pIDs)
50 119	1 066 811	11	97.60% (48916 pIDs)
100 000	2 074 580	11	97.57% (97566 pIDs)
199 527	4 064 816	11	97.54% (194622 pIDs)

Table S8: Upper bound obtained using moment methods on pIDs required, and corresponding minimum design length, to ensure 0.9 probability of 95% good labelling for selected values of RNA molecule count. The rightmost column holds the best quality of labelling observed in 90% of 1000 simulations of labelling with the corresponding RNA molecule counts and numbers of pIDs.

while the latter requires at least a length 15 primer ID (a design including 14 Ns and 1 R, for example, will suffice).

Table S8 shows the bounds obtained using our inexact moment method. Note that these bounds are rigorously derived from model assumptions and easy to calculate, but they are not necessarily very tight: even ignoring the fact that our search algorithm does not guarantee an optimal solution, Chebyshev's inequality does not in general give tight bounds. This is evident in the first two rows of the table. We found that 36 196 pIDs will guarantee 95% good labelling of 1000 RNA molecules, but table S7 already tells us that 97.5% good labelling of the same 1000 RNA molecules can be achieved with fewer pIDs. Likewise, these bounds told us that 60 407 primer IDs will guarantee 95% good labelling of 1995 RNA molecules, but we already knew from table S7 that we can achieve better than 98% good labelling of these 1995 RNA molecules with 60 938 primer IDs. To further illustrate the looseness of our bounds, for each row in table S8, we simulated 1000 trials labelling that number of RNA molecules using that number of pIDs to see what quality of labelling is actually achieved. In all cases, better than 97.5% good labelling was achieved in the sense that a higher number of pIDs were used in at least 90% of the trials. Figure S1 shows histograms of the simulation results for RNA molecule counts of 1000, 10 000, and 100 000, with the corresponding bounds on numbers of pIDs required.

Nonetheless, the results were encouraging, as we saw that achieving 95% good labelling of amplicon pools with a reported RNA molecule count of 10 000 is feasible with primer ID designs of length 10 ($4^{10} = 1\,048\,576$ possible primer IDs), and achieving 95% good labelling of amplicon pools with a reported RNA molecule count of 100 000 may be possible if a primer ID design of length 11 ($4^{11} = 4\,194\,304$ possible primer IDs) is acceptable.

S3 Shallow read depths

S3.1 Supplemental materials and methods

S3.1.1 HIV experiment

The processing pipeline performs the following procedure.

- Sequences are aligned against the primer sequence to identify the boundary between the sequencing "preamble" formed by the sequencing tag and primer ID and the actual sequencing template, i.e. the portion that is a DNA copy of some RNA molecule. Sequences which align poorly with the primer sequence are rejected.
- The first ten characters of the preamble are taken to be the sequencing tag. Sequences whose tag does

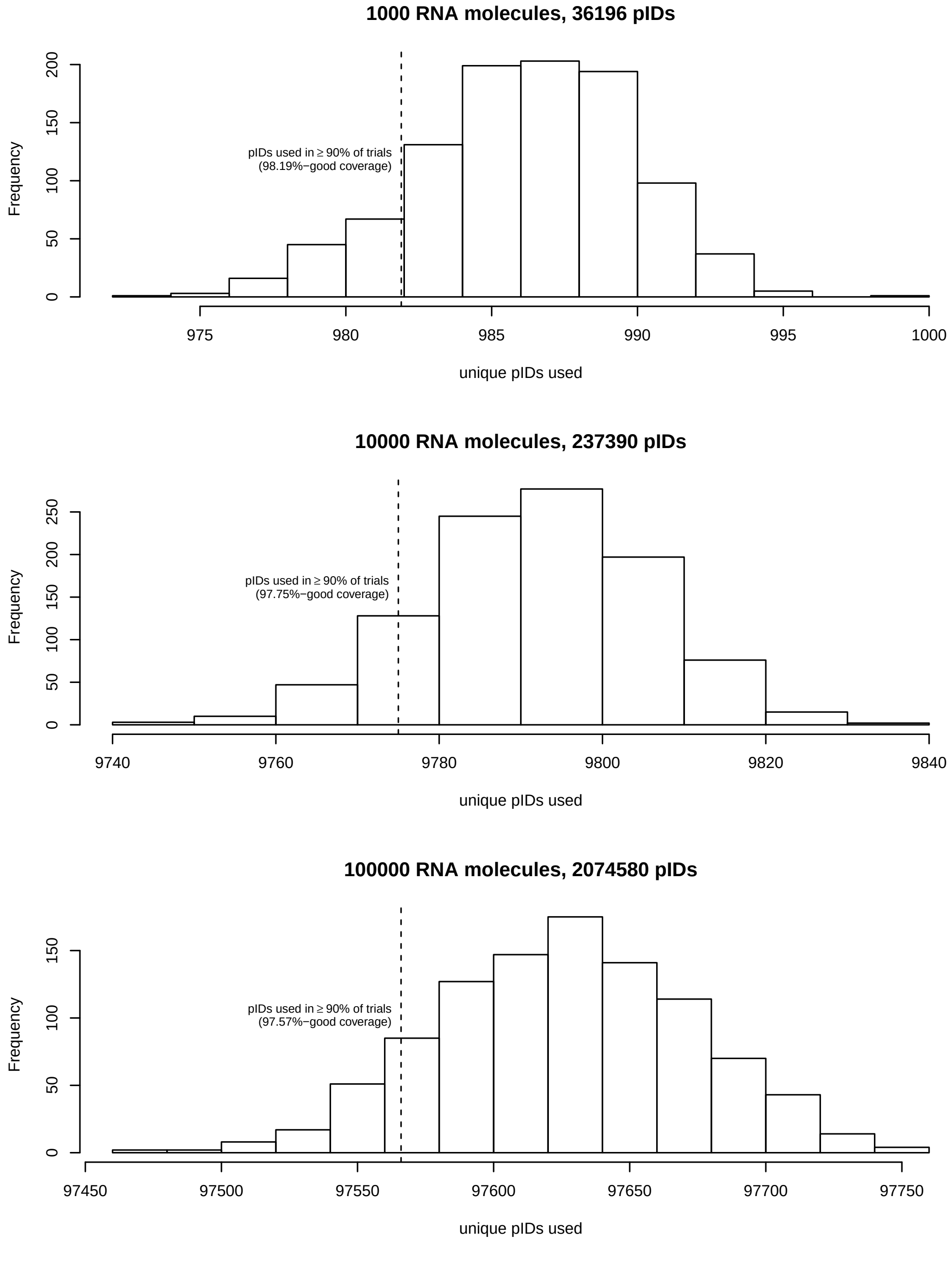

Figure S1: Results of labelling simulations for RNA molecule counts of 1000, 10 000, and 100 000 using our upper bounds on numbers of pIDs required for 95% labelling. The x-axis represents the number of unique pIDs used in a trial. In each case, better than 97.5% good labelling was obtained empirically.

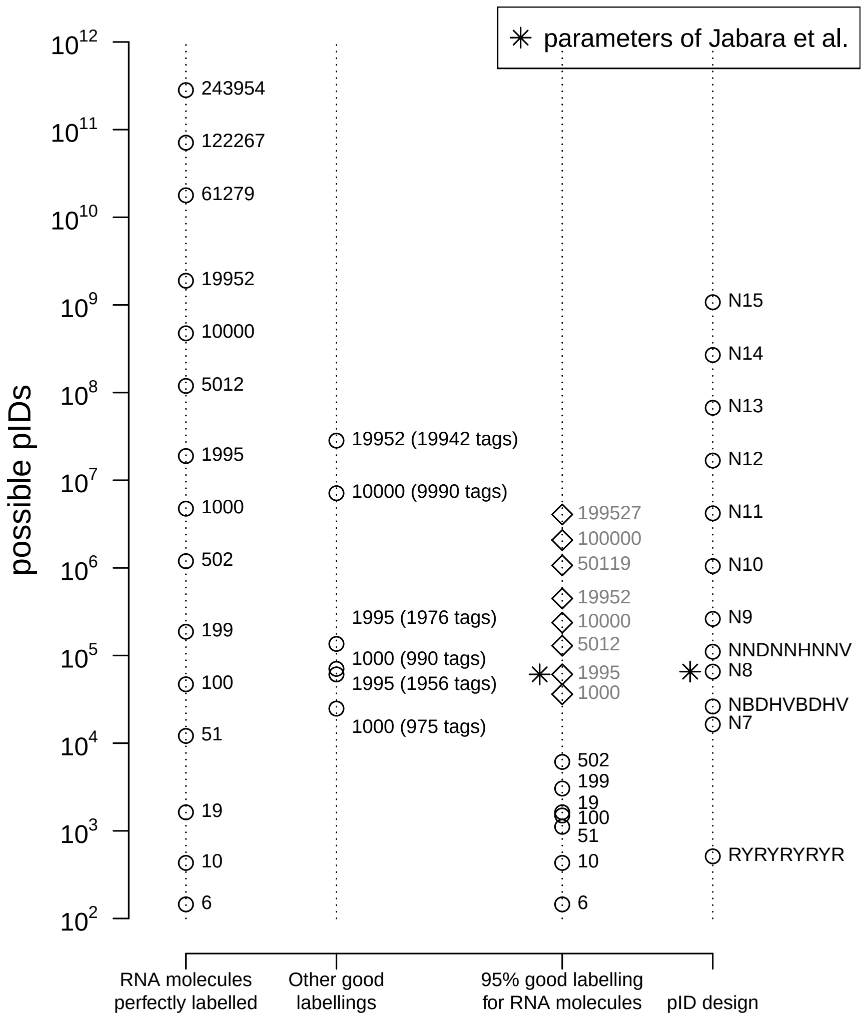

Figure S2: Summary of all of our findings, with the numbers of possible pIDs provided by different designs included. The numbers marked with diamonds in the 95% good labelling column are those obtained via rough moment methods. Note again that as explained in table S6 it takes more pIDs to provide 95% good labelling for 19 RNA molecules than for 100 RNA molecules. The pID design N8, denoted with a star, represents the design used in [1], and the star on the third column represents an estimate of the number of RNA molecules represented in the amplicon pools used in the same.

By design

Design	Good reads	Reads lost	Percentage
NBDHVBDHV	17025	268	1.55
NNDNNHNNV	15411	887	5.44
NNNNNNNNN	18168	827	4.35
RYRYRYRYR	872	10	1.13

By subject and dilution

Subject	Dilution	Good reads	Reads lost	Percentage
P1	1	10289	467	4.34
P2	1	4706	290	5.80
P1	10	6807	566	7.68
P2	10	3684	218	5.59
P1	100	16717	264	1.55
P2	100	9273	187	1.98

Table S9: Percentages of total reads lost due to an improper-length pID, stratified first by primer ID design, and then by subject and dilution. The percentages are computed as $100 \times (\text{reads lost})/(\text{good reads} + \text{reads lost})$.

not match a valid sequencing tag are rejected. The remainder of the preamble is taken to be the pID. We group sequences by their sequencing tag and proceed for each tag individually.

- Recovered primer IDs of length 9 are considered to be real pIDs, free of sequencing error. (Note that this cannot be guaranteed, as there could be sequencing issues in the pID, the tag, or the primer; however, we consider this to be a reasonable policy.) Sequences sharing such a pID are binned together as belonging to the same RNA molecule. After all such pIDs were identified, sequences with pIDs of length 8 or 10 that could be obtained from an already-identified length 9 pID with a single deletion or insertion respectively are recorded and placed into that RNA molecule's bin. Note that if such a pID of length 8 or 10 can be so corrected to more than one proper-length pID, one of the candidate proper-length pIDs is chosen arbitrarily. Any other sequences are rejected.
- RNA molecules with read depth less than three are logged and rejected.

The above alignment procedures are necessary in part due to the 454 platform's difficulties with homopolymer sequencing. The numbers of reads lost due to an improper-length pID may be found in table S9. We use this number as an estimate of the number of reads lost due to indels in the sequencing of the pID, but note that reads may also be lost if the pID itself is of the wrong length due to errors in primer synthesis, or if the pID is incorrectly identified by the alignment procedure. In total, across all sequenced batches, 3.726% of reads were lost.

Binning pIDs of length 8 and 10 with pIDs of proper length that are one insertion or deletion away is a concession to the tendency of the 454 platform to mis-read homopolymers; we reasoned that a pID of length 8 or 10 is more likely to be a misread of a proper-length pID than a poorly-synthesized primer, though we could not be certain of this. Because this binning makes an arbitrary choice of which proper-length pID to resolve to when there is more than one candidate, the attached sequence may be binned with sequences originating from a different RNA molecule. Such corrections were merely intended as a quick-and-dirty way to get a feel for the sequencing error rate of the pIDs. Ultimately, only 639 reads of a total 51 476 reads were so salvaged (1.2 %).

Aside from looking at the primer and sequencing tag portion of the reads, and of course the standard 454 quality filters, our pipeline does relatively little in the way of quality checks on the rest of the read, i.e. the portion copied from the RNA molecule of interest, as its goal is primarily to look at the pID

NBDHVBDHV

position	1 (N)	2 (B)	3 (D)	4 (H)	5 (V)	6 (B)	7 (D)	8 (H)	9 (V)
A	790	**5**	1172	1273	1221	**2**	1172	1282	1287
C	958	1163	**10**	1363	1386	1031	**10**	1243	1336
G	1170	1423	1420	**11**	1598	1466	1377	**10**	1587
T	1303	1630	1619	1574	**16**	1721	1662	1686	**11**
N						1			

NNDNNHNNV

position	1 (N)	2 (N)	3 (D)	4 (N)	5 (N)	6 (H)	7 (N)	8 (N)	9 (V)
A	1726	1780	2294	1739	1725	2414	1789	1850	2579
C	1980	1814	**14**	1951	1910	2453	2021	1894	2725
G	2086	2212	2690	2141	2007	**12**	2045	2025	2767
T	2287	2273	3081	2248	2437	3200	2224	2310	**8**

N9

position	1 (N)	2 (N)	3 (N)	4 (N)	5 (N)	6 (N)	7 (N)	8 (N)	9 (N)
A	837	857	847	938	902	927	952	941	928
C	989	1021	1008	972	985	942	976	910	858
G	1181	1218	1257	1274	1241	1241	1208	1268	1207
T	1342	1252	1237	1165	1221	1239	1213	1230	1356
N		1							

RYRYRYRYR

position	1 (R)	2 (Y)	3 (R)	4 (Y)	5 (R)	6 (Y)	7 (R)	8 (Y)	9 (R)
A	196	**0**	170	**0**	181	**0**	181	**0**	149
C	**1**	152	**0**	143	**0**	177	**1**	168	**0**
G	176	**0**	203	**0**	192	**0**	191	**0**	224
T	**0**	221	**0**	230	**0**	196	**0**	205	**0**

Table S10: Nucleotide composition of pIDs by position. The prohibited nucleotides at each position are emphasized in the tables. An N appearing in a pID means that the sequencer could not discern the base, not that it is actually a degenerate base.

itself. We compared it to the pipeline used for our HCV data (described in section S3.1.2); this pipeline processed the tag-pID-primer section differently and also did several other quality checks, including some that considered the rest of the read. We found that both pipelines produced similar results: when testing new implementations of each on the same data, they gave concordant results on 55 534 reads and discordant results on only 1634 reads (2.86%).

Primer synthesis errors may also affect our results. As mentioned above, deletions during primer synthesis would lead to the resulting pID being discarded during processing, or possibly to being binned with another pID of proper length. Misincorporations (i.e. pIDs which do not match their design, such as one which contains a C in a position where the design specifies a D) might potentially affect the results, but note that our pipeline does not reject pIDs which do not match their design, so such reads would still be retained. Table S10 shows the nucleotide compositions of our different primer ID designs by position; it suggests that by and large our pIDs were well-synthesized, and that misincorporations likely did not substantially affect any of our results.

S3.1.2 HCV experiment

Where the pipeline used for our HIV experiment was geared toward counting as many distinct molecules (and therefore pIDs) as possible, the processing pipeline used for the HCV experiment was developed with the goal of producing clean sequence data (the experiment used pIDs primarily as a means to correct for PCR bias). This processing pipeline performs the following procedure.

- Sequences are inspected to find the sequencing barcode. Those that do not match a sequencing barcode exactly are rejected.
- The first 55 characters of each remaining sequence are subsequently aligned against a sequence composed of the sequence's barcode, nine wild-card characters intended to capture the pID, and the sequencing primer used (in the case of our HCV experiment, this is the DM100 primer), in the reverse read case; in the forward case, the nine wild-card characters are omitted. Sequences for whom this alignment is too poor are rejected. For reverse reads, the pID is taken to be the chunk which matches the nine-wild card characters; for forward reads, the pID is searched for in the last 100 characters of the sequence by looking for the (reverse-complemented) sequencing barcode, and then taken as the 9 bases adjoining it. Note, though, that our HCV data was entirely composed of reverse reads. The sequence is now "clipped" to remove the tag-pID-primer preamble and reverse-complemented if appropriate (i.e. for reverse reads), although the original sequence is retained.
- The next step is to inspect the clipped sequence for spurious occurrences of the Roche QC primer, which is indicative of problems in the amplification chemistry.
- A check on the length of the clipped sequence follows, eliminating reads that are too short.
- The remaining sequences are aligned against a standard to eliminate sequences that are too far from what is expected.
- Lastly, the sequences that survive the last filter are checked for suitable overlap in their alignment against the standard; that is, that a suitable number of bases in the alignment are matches.

Note that, unlike the pipeline used for our HIV experiment, no binning of improper-length pIDs with proper-length pIDs occurs in this pipeline. Because such a binning may potentially group reads stemming from different RNA molecules, it runs counter to the goal of this pipeline, which is to provide clean sequence data for analysis.

S3.2 Theoretical model

The statistical model we consider is as follows. Suppose that after reverse transcription, an amplicon pool represents m RNA molecules; assume that all have been uniquely labelled by pIDs and are thus distinguishable from each other even if the virus RNA molecules they come from are identical. Assume that amplification is unbiased, so that all RNA molecules are represented by equal proportions of copies in the amplicon pool. Therefore the resulting amplicon pool is effectively an infinite "soup" comprised of m components in equal proportions. Now, we draw N amplicons uniformly at random and read their sequences, and we number the reads $1, 2, \ldots, N$. Define R_1 to be the number of singleton RNA molecules represented in the reads. Since each such singleton must be represented by a single read,

$$R_1 = \sum_{j=1}^{N} \mathbf{1}\{\text{read } j \text{ represents a singleton}\}. \tag{S6}$$

Define R_2 to be the number of doubleton RNA molecules represented in the reads. Any such doubleton must be represented by exactly two reads, so

$$R_2 = \sum_{1 \le i < j \le N} \mathbf{1}\{\text{reads } i \text{ and } j \text{ represent a doubleton}\}. \tag{S7}$$

If processing removes all singletons and doubletons, then given the number of reads N and number of RNA molecules m present in the amplicon pool, we would like to consider the number of resulting RNA molecules that are thrown away, i.e. $R_1 + R_2$. To this end, we use Chebyshev's inequality again to construct a confidence interval for the value of $R_1 + R_2$. This requires computing the expected value and variance of R_1 and R_2, as well as the covariance of R_1 and R_2. These computations, which are performed in section S3.2.1, have a similar combinatorial flavour to those for T_1 in section S2.3, but the calculations should not be confused with each other due to the different meanings of the variables.

With these quantities, we are able to construct crude confidence intervals for $R_1 + R_2$. Let μ and σ be the mean and standard deviation of $R_1 + R_2$ respectively. Chebyshev's inequality tells us that

$$\mathbb{P}\Big\{-\sqrt{20}\sigma \leq R_1 + R_2 - \mu \leq \sqrt{20}\sigma\Big\} \geq \frac{1}{20} = 0.95.$$

Both μ and σ are functions of N and m. By linearity we know that $\mu = \mathbb{E}[R_1] + \mathbb{E}[R_2]$, and we also know that

$$\sigma = \sqrt{\mathrm{var}(R_1 + R_2)} = \sqrt{\mathrm{var}(R_1) + \mathrm{var}(R_2) + \mathrm{cov}(R_1, R_2)}.$$

Given an RNA molecule count m and number of reads N, we can thus compute the above confidence interval. Bear in mind that, as mentioned in the main text, this is not a tight confidence interval, and the probability of $R_1 + R_2$ lying inside it is *at least* 95%; conversely, the probability of $R_1 + R_2$ lying outside it is *at most* 5%.

S3.2.1 Moment calculations for R_1 and R_2

We start by considering R_1. The probability of any read representing a singleton RNA molecule is $((m-1)/m)^{N-1}$. To see this, consider that the read may represent any one of the m ingredients in the soup, which has probability 1. Every other read must represent another ingredient, which has probability $(m-1)/m$; there are $N-1$ other reads, so we take the product of all their probabilities. Thus we find that

$$\mathbb{E}[R_1] = N\left(\frac{m-1}{m}\right)^{N-1}. \tag{S8}$$

To compute the variance we first compute $\mathbb{E}[R_1^2]$. Let $\mathbf{1}_i$ be the indicator function of the event that read i represents a singleton.

$$\begin{aligned} R_1^2 &= \sum_{i=1}^{N}\sum_{j=1}^{N} \mathbf{1}_i \mathbf{1}_j \\ &= \sum_{i=1}^{N} \mathbf{1}_i + \sum_{1\leq i<j\leq N} \mathbf{1}_i \mathbf{1}_j, \end{aligned} \tag{S9}$$

because on the diagonal terms $i = j$ we have $\mathbf{1}_i\mathbf{1}_j = \mathbf{1}_i$. The first sum on the last line is simply R_1. In the last sum on the last line, $\mathbf{1}_i\mathbf{1}_j$ is equal to the indicator of the event that both reads i and j represent singletons. This has probability

$$\frac{m-1}{m}\left(\frac{m-2}{m}\right)^{N-2},$$

because read i can represent any RNA molecule (probability 1), read j must choose any other RNA molecule (probability $(m-1)/m$), and every other particle must choose an RNA molecule other than the two picked by i and j (probability $(m-2)/m$ each); multiply all of these factors together to get the result. Using this in equation (S9), we can compute that

$$\mathbb{E}\big[R_1^2\big] = N\left(\frac{m-1}{m}\right)^{N-1} + \binom{N}{2}\frac{m-1}{m}\left(\frac{m-2}{m}\right)^{N-2}.$$

Thus combining this and equation (S8), we obtain

$$\begin{aligned}\operatorname{var}(R_1) &= \mathbb{E}\big[R_1^2\big] - \mathbb{E}[R_1]^2 \\ &= N\left(\frac{m-1}{m}\right)^{N-1} + \binom{N}{2}\frac{m-1}{m}\left(\frac{m-2}{m}\right)^{N-2} \\ &\quad - N^2\left(\frac{m-1}{m}\right)^{2N-2}.\end{aligned}$$

We now turn our attention to R_2. The probability that reads i and j represent a doubleton pID is

$$\frac{1}{m}\left(\frac{m-1}{m}\right)^{N-2}.$$

To see this, imagine that read i chooses an RNA molecule arbitrarily; then read j must choose the same RNA molecule (probability $1/m$); then every other read must choose another RNA molecule (probability $(m-1)/m$ each). Thus

$$\mathbb{E}[R_2] = \binom{N}{2}\frac{1}{m}\left(\frac{m-1}{m}\right)^{N-2}.$$

We compute the variance of R_2 by first computing $\mathbb{E}[R_2^2]$, as we did for R_1. Let $\mathbf{1}_{ij}$ be the indicator of the event that the read i and j represent a doubleton RNA molecule. We have

$$\begin{aligned}R_2^2 &= \sum_{1\le i<j\le N}\ \sum_{1\le k<l\le N} \mathbf{1}_{ij}\mathbf{1}_{kl} \\ &= \sum_{\substack{1\le i<j\le N \\ 1\le k<l\le N \\ i,j,k,l \text{ all distinct}}} \mathbf{1}_{ij}\mathbf{1}_{kl} + \sum_{1\le i<j\le N} \mathbf{1}_{ij}.\end{aligned} \tag{S10}$$

The last sum comes from terms where $i = k$ and $j = l$. Any term with exactly one of i, j equalling exactly one of k, l disappears: this is because, for example, if $i = k$, then it is impossible for (i, j) and (k, l) to both represent doubletons, as if that were the case then all of reads i, j, and l would have to share an RNA molecule, making (i, j, l) a tripleton.

Supposing that $i < j$, $k < l$, and all of i, j, k, l are distinct, the probability of the event that both (i, j) and (k, l) represent doubletons is

$$\frac{m-1}{m^3}\left(\frac{m-2}{m}\right)^{N-4}.$$

First, read i is free to choose any RNA molecule (probability 1). Read j must choose the same RNA molecule as read i (probability $1/m$). Read k can choose any other RNA molecule (probability $(m-1)/m$); read l chooses the same one as k (probability $1/m$). Every other read has $m-2$ possible options (probability $(m-2)/m$ each). Plugging this into equation (S10), and noting that the first sum on the last line has

$$\binom{N}{2}\binom{N-2}{2}$$

summands, we get

$$\mathbb{E}\big[R_2^2\big] = \binom{N}{2}\binom{N-2}{2}\frac{m-1}{m^3}\left(\frac{m-2}{m}\right)^{N-4} + \binom{N}{2}\frac{1}{m}\left(\frac{m-1}{m}\right)^{N-2}.$$

Thus

$$
\begin{aligned}
\operatorname{var}(R_2) &= \mathbb{E}\left[R_2^2\right] - \mathbb{E}[R_2]^2 \\
&= \binom{N}{2}\binom{N-2}{2}\frac{m-1}{m^3}\left(\frac{m-2}{m}\right)^{N-4} + \binom{N}{2}\frac{1}{m}\left(\frac{m-1}{m}\right)^{N-2} \\
&\quad - \binom{N}{2}^2\frac{1}{m^2}\left(\frac{m-1}{m}\right)^{2N-4}.
\end{aligned}
$$

The last ingredient we need for our analysis is $\operatorname{cov}(R_1, R_2)$. To this end, we first compute $\mathbb{E}[R_1R_2]$.

$$
\begin{aligned}
R_1R_2 &= \sum_{i=1}^{N} \mathbf{1}_i \sum_{1\le j<k\le N} \mathbf{1}_{jk} \\
&= \sum_{i=1}^{N} \sum_{\substack{1\le j<k\le N \\ j,k\ne i}} \mathbf{1}_i\mathbf{1}_{jk}.
\end{aligned}
$$

The terms where $i = j$ or $i = k$ vanish because i cannot represent both a singleton and a doubleton. Each summand is the indicator of the event that i represents a singleton and (j, k) represents a doubleton; this has probability

$$
\frac{m-1}{m^2}\left(\frac{m-2}{m}\right)^{N-3}.
$$

To see this, consider that read i is free to choose any RNA molecule (probability 1); j must choose any other RNA molecule (probability $(m-1)/m$) and k must choose the same RNA molecule as j (probability $1/m$); and every other read thus has $m-2$ options to choose from (probability $(m-2)/m$ each). There are

$$
N\binom{N-1}{2}
$$

summands to consider, because if any i is chosen to be the singleton, there are $N-1$ remaining reads from which to choose two to become the doubleton. Thus

$$
\mathbb{E}[R_1R_2] = N\binom{N-1}{2}\frac{m-1}{m^2}\left(\frac{m-2}{m}\right)^{N-3},
$$

and so

$$
\begin{aligned}
\operatorname{cov}(R_1, R_2) &= \mathbb{E}[R_1R_2] - \mathbb{E}[R_1]\mathbb{E}[R_2] \\
&= N\binom{N-1}{2}\frac{m-1}{m^2}\left(\frac{m-2}{m}\right)^{N-3} \\
&\quad - N\binom{N}{2}\frac{1}{m}\left(\frac{m-1}{m}\right)^{2N-3}.
\end{aligned}
$$

S3.3 Results: supplemental details

S3.3.1 HIV experiment

Figures S3 and S4 show the observed numbers of singletons and doubletons for each sequenced batch. Some batches appear twice; this is because these batches were sequenced twice, on two different plate regions. The total number of observed RNA molecules for each batch is plotted with a triangle; it is evident how large a proportion of the RNA molecules were singletons and doubletons.

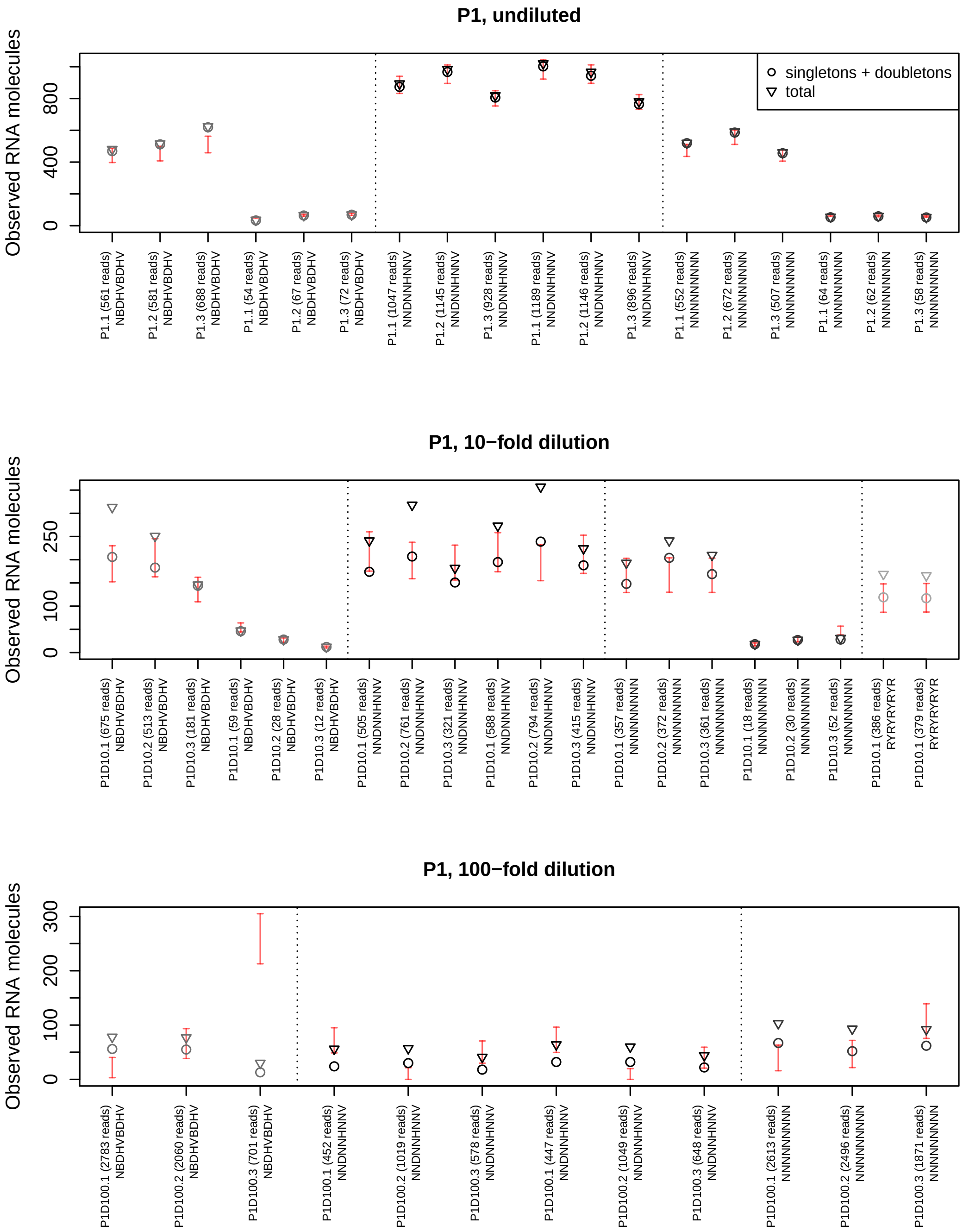

Figure S3: Number of singleton/doubleton RNA molecules experimentally observed in batches coming from subject P1 for the three dilution factors (circles). The triangles are the total number of RNA molecules observed in each batch. For each batch, the number of reads produced in sequencing is listed in the label on the x-axis. The bars are loose theoretically-predicted 95% confidence intervals for the number of singletons/doubletons, based on the assumption that the RNA molecule count is $m = \lceil(\text{lab efficiency} \times \text{rVL})/(24 \times \text{dilution factor})\rceil$, where the lab efficiencies for the different extract-dilution-design combinations are given in table S12.

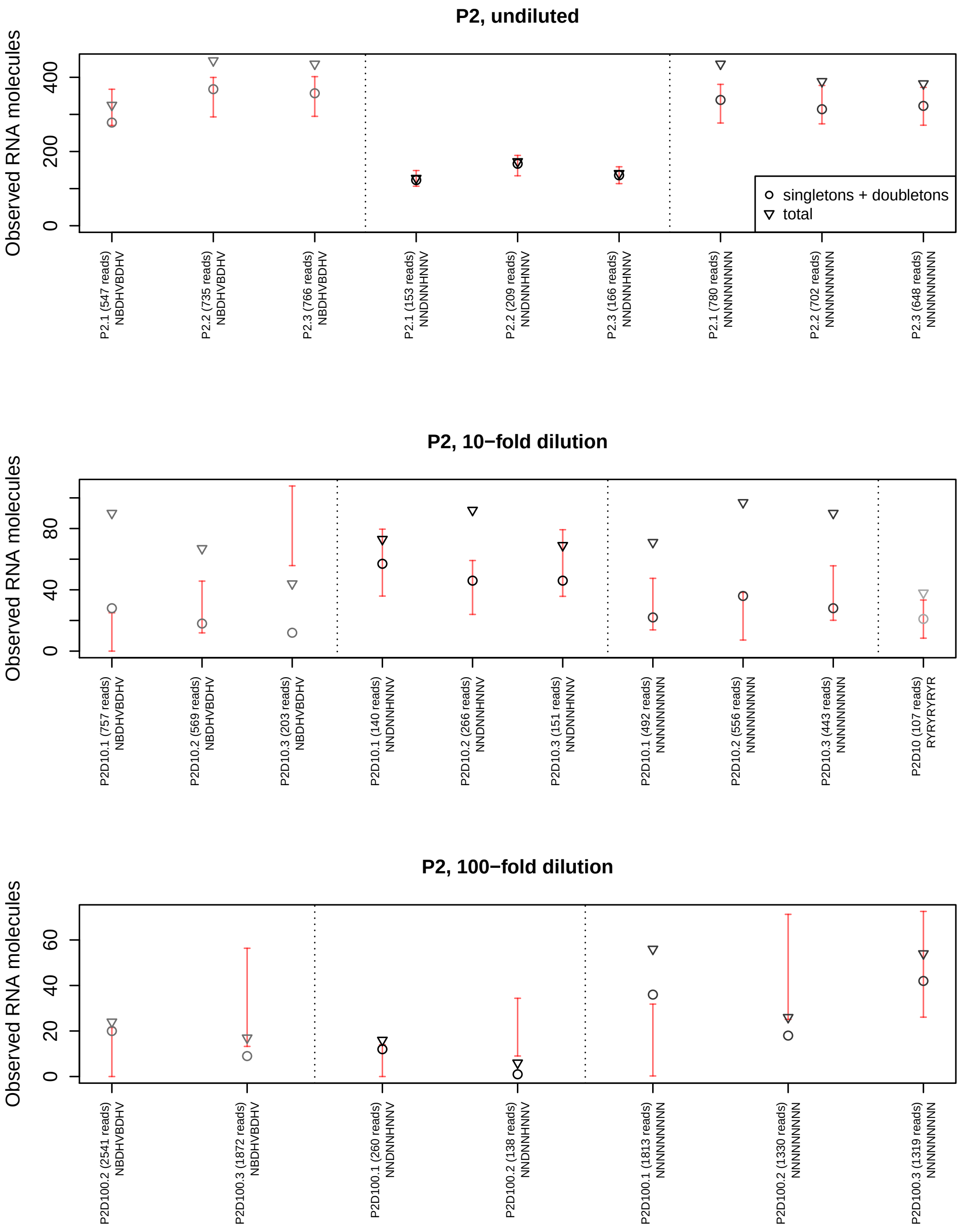

Figure S4: Analogous plots to figure S3 for subject P2.

By pID design

Design	Total reads	Singleton reads	Doubleton reads
NBDHVBDHV	17025	2920 (17.15%)	1358 (7.98%)
NNDNNHNNV	15411	6114 (39.67%)	2282 (14.81%)
NNNNNNNNN	18168	2971 (16.35%)	1374 (7.56%)
RYRYRYRYR	872	151 (17.32%)	212 (24.31%)

By subject and dilution

Subject	Dilution	Total reads	Singleton reads	Doubleton reads
P1	1	10289	7882 (76.61%)	1928 (18.74%)
P2	1	4706	1722 (36.59%)	1366 (29.03%)
P1	10	6807	1813 (26.63%)	1580 (23.21%)
P2	10	3684	232 (6.30%)	164 (4.45%)
P1	100	16717	402 (2.40%)	122 (0.73%)
P2	100	9273	105 (1.13%)	66 (0.71%)

Table S11: Number and percentage of reads belonging to singleton or doubleton pIDs, stratified first by pID design, and then by subject and dilution.

The confidence intervals were produced using Chebyshev's inequality as described in section S3.2, taking the RNA molecule count m to be

$$m = \frac{(\text{lab efficiency})(\text{rVL})}{24(\text{dilution factor})}$$

and N to be the observed number of reads produced by sequencing and passing all filtering stages. The lab efficiency factor takes into account that the number of RNA molecules is affected by several factors in the amplification chemistry, such as RNA extraction and RT efficiency. Moreover, it also accounts for errors in the viral load assay, which has an error of $0.3\log_{10}$ copies/ml.

This lab efficiency factor is unknown and must be estimated. Because we do not have a likelihood function to maximize, we instead chose it using a heuristic approach. For a given extract-dilution-design combination, we chose the lab efficiency factor to minimize the sum of squared distances between the observed number of singletons and doubletons and the theoretical mean, in units of theoretical standard deviations. This was done using the DEoptim package in R [4, 5, 6, 7, 8]. The values found are in table S12.

The theoretical predictions obtained using these estimated lab efficiency factors appear to at least follow the shape of the true observed data for the undiluted batches, and performed progressively worse as the dilution factor increased. For the 100-fold diluted batches, the model's predictions were admittedly poor. Recall that the confidence intervals found are conservative; thus, the fact that the observed data were so frequently outside these intervals for the 100-fold diluted batches indicates that the model is a poor fit for this data. Figure S5 shows the model's performance degradation using three comparisons of the empirical read depth distribution and the expected read depth distribution for subject P1 using primer ID design NNDNNHNNV: one for each dilution level. The expected distributions were computed using the lab efficiency factors in table S12 to estimate the RNA molecule counts of each batch. It should be noted that in the 100-fold dilution case, the plot does not include pIDs of read depth greater than 10; such pIDs make up a large majority of the total pIDs in both the theoretical and empirical distributions.

These discrepancies between dilutions could be explained in several ways. For one, inaccurate dilution may have contributed to the lower agreement at higher dilutions. Moreover, the model does not explicitly consider reverse transcription as a stochastic process, which could greatly affect the number of RNA molecules that successfully amplify; such a stochastic process may be affected by the dilution. The RT efficiency may also vary depending on the viral load of the subject (and, it stands to reason, on the dilution factor) [9]. Our estimates in table S12 may be indicative of such a dependence of RT efficiency on viral load.

There are other possible sources of error that are not dilution-specific but should be noted. RNA extrac-

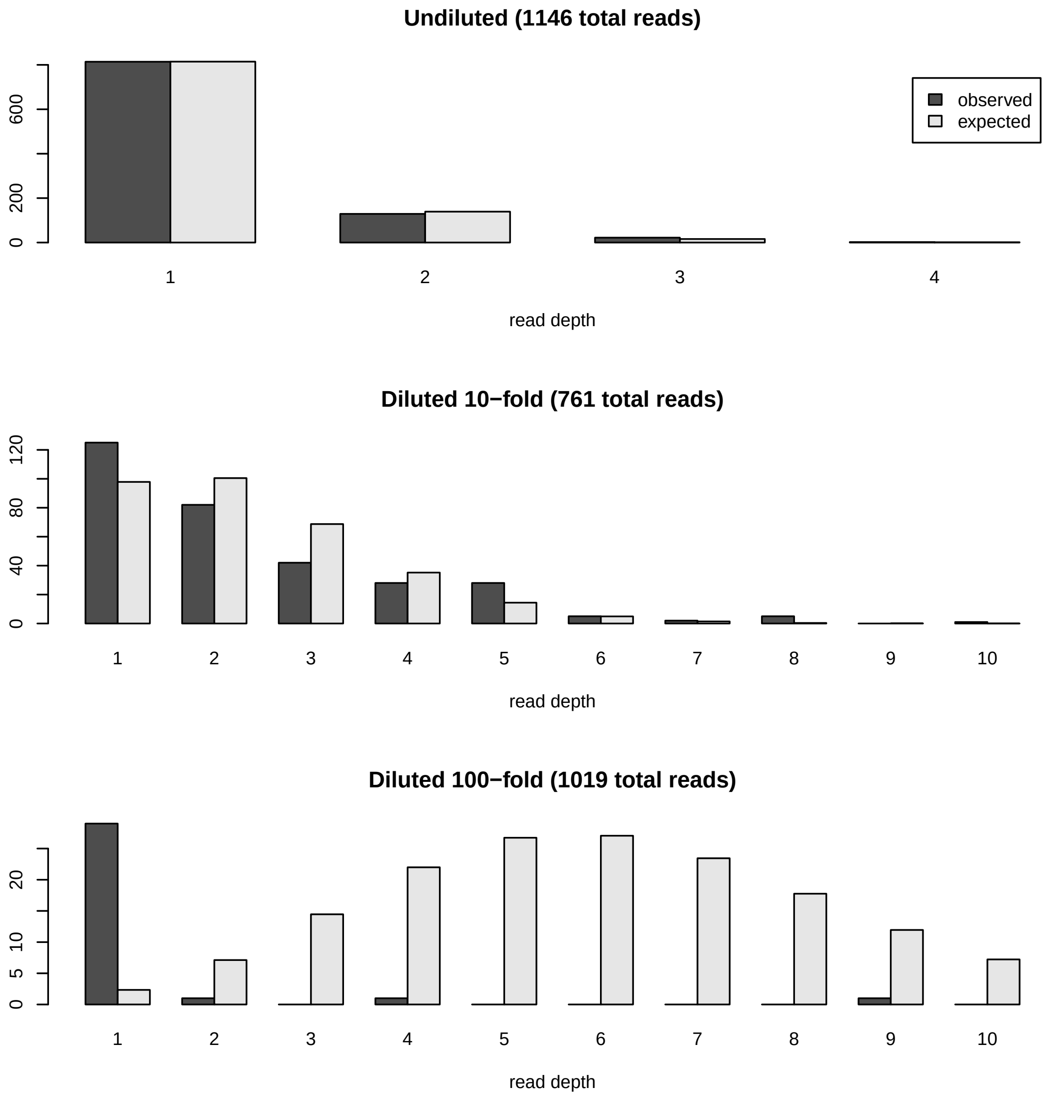

Figure S5: Comparison of the observed and expected read depth distributions for randomly-chosen batches representing subject P1 using primer ID design NNDNNHNNV at each of the three dilution levels. In the undiluted case, the observed number of pIDs with read depth strictly greater than 4 was 179, versus an expected number of 175.2893; in the 10-fold diluted case, the observed number of pIDs with read depth strictly greater than 10 was 443, versus an expected number of 437.5745; and in the 100-fold diluted case, the observed number of pIDs with read depth strictly greater than 10 was 987, versus an expected number of 858.9227.

Subject P1		
Dilution	Design	Lab efficiency
1	NNDNNHNNV	0.26
1	NNNNNNNNN	0.15
1	NBDHVBDHV	0.099
10	NNDNNHNNV	0.29
10	NNNNNNNNN	0.22
10	NBDHVBDHV	0.27
10	RYRYRYRYR	0.16
100	NNDNNHNNV	1.3
100	NNNNNNNNN	3.6
100	NBDHVBDHV	3.4

Subject P2		
Dilution	Design	Lab efficiency
1	NNDNNHNNV	0.061
1	NNNNNNNNN	0.076
1	NBDHVBDHV	0.08
10	NNDNNHNNV	0.13
10	NNNNNNNNN	0.17
10	NBDHVBDHV	0.19
10	RYRYRYRYR	0.059
100	NNDNNHNNV	0.69
100	NNNNNNNNN	4
100	NBDHVBDHV	4.8

Table S12: Estimated lab efficiency factors. This factor is meant to account for the ways that variances in chemistry, lab conditions, and errors in the viral load assay may affect the RNA molecule count of an amplicon pool.

tion may cause discrepancies, as the extractions performed on the two subjects' samples may have differed in efficiency. In the amplification chemistry, bias may have affected the reverse transcription or amplification stages. Sequencing error or data processing errors may have caused different primer IDs to be read as the same. Our theoretical model makes the assumptions that primer IDs bind to RNA molecules uniformly at random during reverse transcription, and that DNA molecules are chosen to be read uniformly at random, which may be unrealistic. If these assumptions did not hold, more molecules may have been completely lost (in the sense of never having been sequenced) than expected, as RNA molecules that were more likely to be tagged with pIDs might have crowded out those that were less likely, and those that yielded DNA molecules more likely to be read than others might similarly have crowded out those producing DNA molecules that were less likely to be read.

It is worth noting that the estimated lab efficiency factors for the RYRYRYRYR design for both subjects were smaller than those of the other designs at the same dilution factor. We believe that this is evidence of another factor: the labelling of RNA molecules with pIDs. The fact that different RNA molecules may well share a pID can cause fewer singletons and doubletons to be identified by the processing, as several RNA molecules that only appear once or twice may be merged by their pID, resulting in a primer ID with more than two associated reads coming from different RNA molecules. We know from figure S2 that the 512 possible pIDs provided by this design would not be enough to ensure 0.9 probability of 95% good labelling for amplicon pools with RNA molecule counts in the range of 163 (e.g. an amplicon pool produced from subject P1 at 10 times dilution, factoring in a 0.22 lab efficiency as estimated for the N9 primer design) or 169 (e.g. an amplicon pool produced from subject P2 at the same 10 times dilution, factoring in a 0.13 lab efficiency as estimated for the NNDNNHNNV design). Thus inadequate labelling may have had a substantial effect on these batches, and the lower estimated lab efficiency for the batches run with primer ID design RYRYRYRYR, P1D10.1 and P2D10, may have been at least in part due to it, as this lower estimate caused the theoretically predicted values to be smaller (that is, the error bars on the plots in figures S3 and S4 are lower down) so that they better match the observed data.

S3.3.2 HCV experiment

Unlike in the HIV experiment, all sequenced batches in the HCV experiment used the same primer ID design (N9), came from different subjects, and were intended to have the same RNA molecule count, with 10 000 molecules entering the RT reaction. When producing plots analogous to figures S3 and S4, we initially used an RNA molecule count of 10 000, believing this to be an overestimate due to attrition in the RT step. This was immediately problematic because there are more than this number of unique pIDs observed in many of the batches. This produced confidence intervals for the number of singletons and doubletons that were in

most cases not at all close to the observed. While this may have been indicative of issues in the sequencing of pIDs (that is, there could have been 10 000 or fewer pIDs but in sequencing some were mis-read as other ones), or of unsuitability of our model, we suspected that this was in large part an issue of an incorrect estimate of the RNA molecule count.

To compensate for this, we used a different means of estimating the RNA molecule count for our HCV experiment data. Using the same model, for each batch we produced a maximum likelihood estimate of the RNA molecule count using the number of reads produced for that batch and the total number of pIDs observed (as a proxy for the total number of RNA molecules represented in the reads). We then used this estimate to produce the confidence intervals in the plot in the same fashion as above. Figure S6 shows this plot. Note in the figure how widely the number of observed RNA molecules varies, strongly indicative that a fixed estimate of 10 000 RNA molecule count for all of them is likely unsuitable. As with figures S3 and S4, this plot shows that our model produces mixed results (recall that confidence intervals not containing the true number of singletons plus doubletons should be considered evidence of a poor model fit), although it still appears to capture some of the true behaviour. It should be noted that the batch using MID 11, which according to the plot was especially poorly fit by our model, was strongly affected by sampling bias. In this batch, a single pID was observed in over 30 000 reads of a total 48993. Though this was clearly a spurious case for our model, it illustrates the value of using pIDs, as that single RNA molecule was massively overrepresented in the reads and may not have been caught as such without using pIDs.

As in the HIV experiment, it should be noted that the data from our HCV experiment may be affected by biases introduced in extraction, reverse transcription, amplification, sampling, or the like.

S4 Effects of pIDs on amplification and sequencing: microscopic

S4.1 Regressions

The homopolymer score of each observed pID is computed as follows.

- We first consider every homopolymer in the pID. For example, the pID CCTCGAAAC contains the homopolymers CC and AAA.
- To each homopolymer we count the number of *sub-homopolymers* it contains, counting itself as one. In the above example, CC only contains one sub-homopolymer (itself), while AAA contains three: an AA comprised of the first two characters; an AA comprised of the last two characters; and AAA itself. This is akin to considering the homopolymer as a string and counting the number of ways we can choose a start and end position for a substring of length greater than 1, and this has a closed formula: if a homopolymer has length n, then this homopolymer contains $n(n-1)/2$ total sub-homopolymers.
- We sum the counts for each homopolymer to create the pID's homopolymer score. In the above example, the pID gets a score of 4.

In our regressions on the HIV data we consider the following explanatory variables: the homopolymer score of the pID; the number of Cs, Gs, and Ts in the pID (the number of As is implicit given these numbers); the reported viral load of the batch, defined as the rVL of the subject divided by the dilution factor of the batch; the pID design; the sequencing barcode used (twelve barcodes, named A-L – see table S1 for their sequences); and the region of the 454 plate that the batch was run on (1-7). We consider the homopolymer score and the nucleotide content to be *intrinsic* to the pID, and all the other factors to be *extrinsic* – they depend on circumstances outside of the pID such as the size of the batch, the RT and amplification chemistry of the batch's associated primers, and the sequencing platform and whatever irregularities it might introduce.

Our regressions on the HCV data omitted the extrinsic factors, as the setup of the experiment was different in several ways: only one primer ID design was used; only one plate region was used; all batches were intended to represent the same number of RNA molecules; and all batches came from different virus populations, so any effects of the barcodes or of irregularities in the laboratory procedures between batches are confounded by the effects of simply coming from different sources.

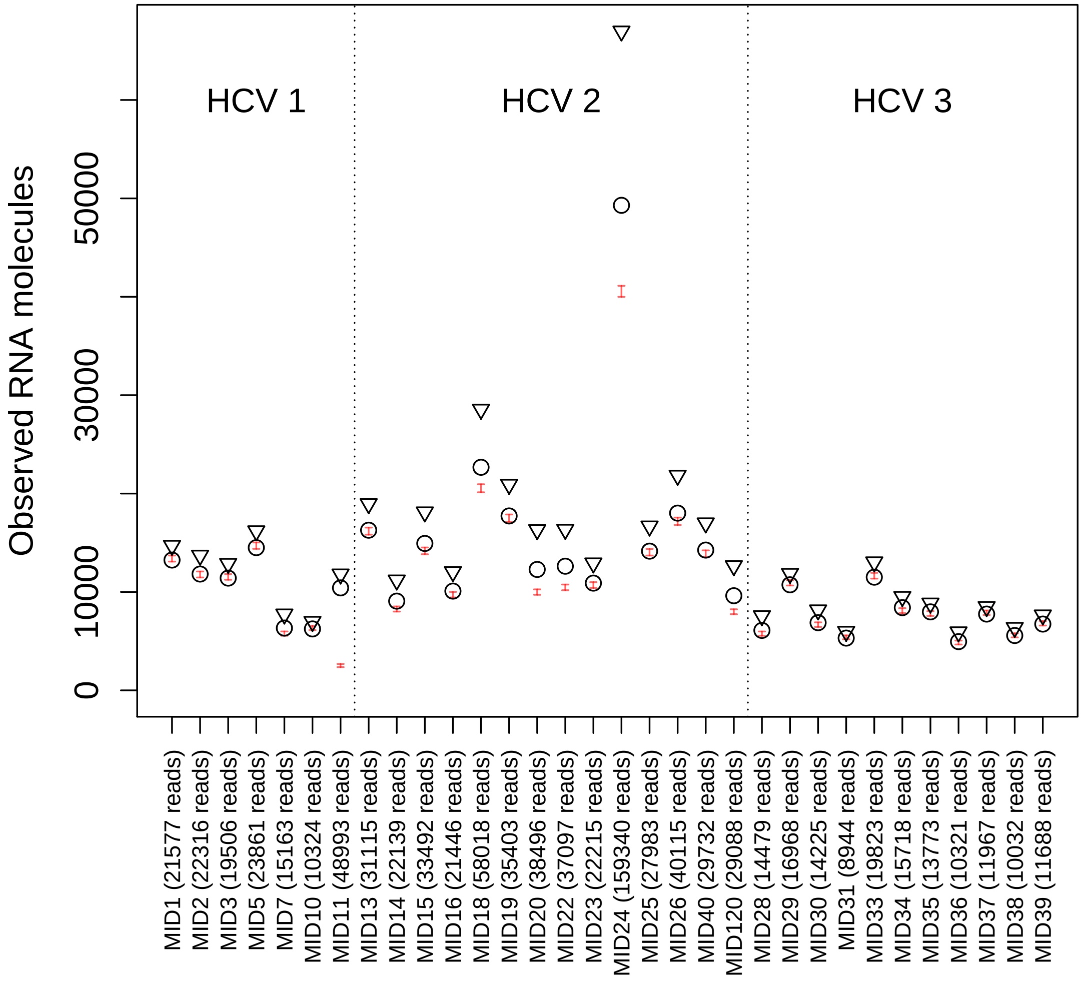

Figure S6: Analogous plots to figure S3 for the HCV experiment. Confidence intervals were generated using the number of reads and estimated RNA molecule count for each batch; this estimate was made using both the number of reads and the total number of pIDs observed.

Regression coefficients for the HIV data may be found in tables S13 and S14; the average quality scores by plate region are in table S15. Regression coefficients for the HCV data may be found in table S16.

S5 Effects of pIDs on amplification and sequencing: macroscopic

Tables S19 and S20 contain the coefficients of the multinomial logistic regressions on the effects of error correction scheme on the prevalences of the top six variants found in subject P1.

Figure S7 is the analogous plot to figure 5 of the main text for the top three variants of P2, and tables S21 and S22 contain the coefficients of our multinomial logistic regression on the effects of error correction scheme on the top three variants found in subject P2. We found that the keep scheme used had significant effects on the prevalences. Removing singletons was associated with increased log odds for all three variants (variant 1, $p < 0.0001$; variant 2, $p = 0.000\,26$; variant 3, $p = 0.030$), and removing both singletons and doubletons had significant associations with increased log odds for variants 1 and 2 (variant 1, $p = 0.000\,19$; variant 2, $p = 0.0073$; variant 3, $p = 0.18$). Meanwhile, changing the mixture resolution scheme from censoring to equal weighting had a significant association with a decreased prevalence of variant 1, and no significant effects on the other variants (variant 1, $p = 0.011$; variant 2, $p = 0.16$; variant 3, $p = 0.25$). Under a keep scheme of retaining all pIDs, 2661 RNA molecules were observed across all P2 batches; when rejecting singletons this number dropped to 1162; and when rejecting singletons and doubletons the number dropped again to 656 (all while censoring mixtures). Changing from a mixture resolution scheme that censored all mixtures to the equal weighting scheme, the number of RNA molecules observed increased from 2661 to 2845.

References

[1] Jabara, C., Jones, C., Roach, J., Anderson, J., and Swanstrom, R. (2011) *Proceedings of the National Academy of Sciences* **108(50)**, 20166–20171.

[2] Murphy, D. G., Willems, B., Deschênes, M., Hilzenrat, N., Mousseau, R., and Sabbah, S. (2007) *Journal of clinical microbiology* **45(4)**, 1102–1112.

[3] 454 Life Sciences Corporation 454 Sequencing Technical Bulletin TCB No. 005-2009 edition April 2009.

[4] Ardia, D., Mullen, K. M., Peterson, B. G., and Ulrich, J. DEoptim: Differential Evolution in R (2012) version 2.2-1.

[5] Mullen, K., Ardia, D., Gil, D., Windover, D., and Cline, J. (2011) *Journal of Statistical Software* **40(6)**, 1–26.

[6] Ardia, D., Boudt, K., Carl, P., Mullen, K. M., and Peterson, B. G. (2011) *The R Journal* **3(1)**, 27–34.

[7] Ardia, D., Arango, J. O., and Gomez, N. G. (2011) *Wilmott Magazine* **55**, 76–79.

[8] Price, K. V., Storn, R. M., and Lampinen, J. A. January 2006 Differential Evolution - A Practical Approach to Global Optimization, Natural ComputingSpringer-Verlag, ISBN 540209506.

[9] Levesque-Sergerie, J., Duquette, M., Thibault, C., Delbecchi, L., and Bissonnette, N. (2007) *BMC Molecular Biology* **8(1)**, 93.

	Estimate	Std. Error	t value	$\mathbb{P}\{> t\}$
(Intercept)	457.4	15.69	29.16	< 0.0001
homopolymer score	0.2931	0.4607	0.64	0.52
C content	0.6991	0.8855	0.79	0.43
G content	1.245	0.8782	1.42	0.16
T content	-0.9125	1.016	-0.90	0.37
batch rVL	5.082×10^{-4}	3.412×10^{-5}	14.89	< 0.0001
pID design				
N9	-316.1	8.717	-36.26	< 0.0001
NBDHVBDHV	-47.95	8.433	-5.69	< 0.0001
RYRYRYRYR	73.62	10.04	7.33	< 0.0001
Sequencing barcodes				
B	0.8559	14.24	0.06	0.95
C	-57.8	13.33	-4.33	< 0.0001
D	-150	11.79	-12.73	< 0.0001
E	-146.9	11.9	-12.35	< 0.0001
F	-145.5	11.83	-12.30	< 0.0001
G	-383.2	15.1	-25.38	< 0.0001
H	-377.1	15.16	-24.87	< 0.0001
I	-386.7	15.15	-25.53	< 0.0001
J	-390.8	16.99	-23.00	< 0.0001
K	-403.9	16.46	-24.54	< 0.0001
L	-420.6	17.11	-24.59	< 0.0001
Plate region				
2	-47.35	6.754	-7.01	< 0.0001
3	240	7.152	33.55	< 0.0001
4	34.13	3.086	11.06	< 0.0001
5	122.3	5.581	21.92	< 0.0001
6	12.74	7.81	1.63	0.10

Table S13: Coefficients of fit of pID "success". The baseline case is for the pID AAAAAAAAA coming from the design NNDNNHNNV and sequenced using barcode A on plate region 1. Note that there is no coefficient associated with the factor representing plate region 7; the fitting routine detected a linear dependence between this and the other explanatory factors, so it was omitted. The fit assumed a gamma distribution with inverse link function, so that the estimated mean of the distribution is the inverse of the linear predictor η defined by the coefficients.

	Estimate	Std. Error	z value	$\mathbb{P}\{>\|z\|\}$
(Intercept)	-4.787	0.3449	-13.88	< 0.0001
homopolymer score	0.1065	0.01136	9.37	< 0.0001
C content	0.02685	0.04017	0.67	0.50
G content	0.07027	0.03618	1.94	0.052
T content	1.983×10^{-5}	0.04025	0.00	1.00
batch rVL	1.484×10^{-6}	7.337×10^{-7}	2.02	0.043
pID design				
N9	2.21	0.3266	6.77	< 0.0001
NBDHVBDHV	2.212	0.3876	5.71	< 0.0001
RYRYRYRYR	-0.0999	0.6027	-0.17	0.87
Sequencing barcodes				
B	-0.2184	0.1827	-1.20	0.23
C	-0.1583	0.1932	-0.82	0.41
D	0.1309	0.1954	0.67	0.50
E	-0.1301	0.2169	-0.60	0.55
F	0.6262	0.1952	3.21	0.0013
G	-0.4752	0.3283	-1.45	0.15
H	-0.4975	0.2955	-1.68	0.092
I	-0.3197	0.3033	-1.05	0.29
J	-0.4943	0.36	-1.37	0.17
K	0.3839	0.3555	1.08	0.28
L	-0.5313	0.489	-1.09	0.28
Plate region				
2	0.7451	0.2541	2.93	0.0034
3	-1.615	0.312	-5.17	< 0.0001
4	-2.318	0.3183	-7.28	< 0.0001
5	-1.731	0.3038	-5.70	< 0.0001
6	0.09122	0.1837	0.50	0.6194

Table S14: Coefficients of logistic regression on pID indel error. As in table S13 there is no coefficient corresponding to the factor representing plate region 7. The intercept gives the log-odds of a pID containing an indel error for the pID AAAAAAAAA coming from design NNDNNNHNNV, sequenced using barcode A on plate region 1. Each factor's coefficient represents the change in log-odds explained by that factor.

Region	Average quality score (SD)
1	34.659 (8.219)
2	30.007 (9.169)
3	34.976 (7.993)
4	36.056 (7.249)
5	34.473 (8.108)
6	34.835 (8.127)
7	27.547 (8.569)

Table S15: Average quality scores of all non-control reads as reported by the 454 for each region. The averages were computed over all bases of reads passing all 454 quality filtering, including those that were rejected by the processing pipeline.

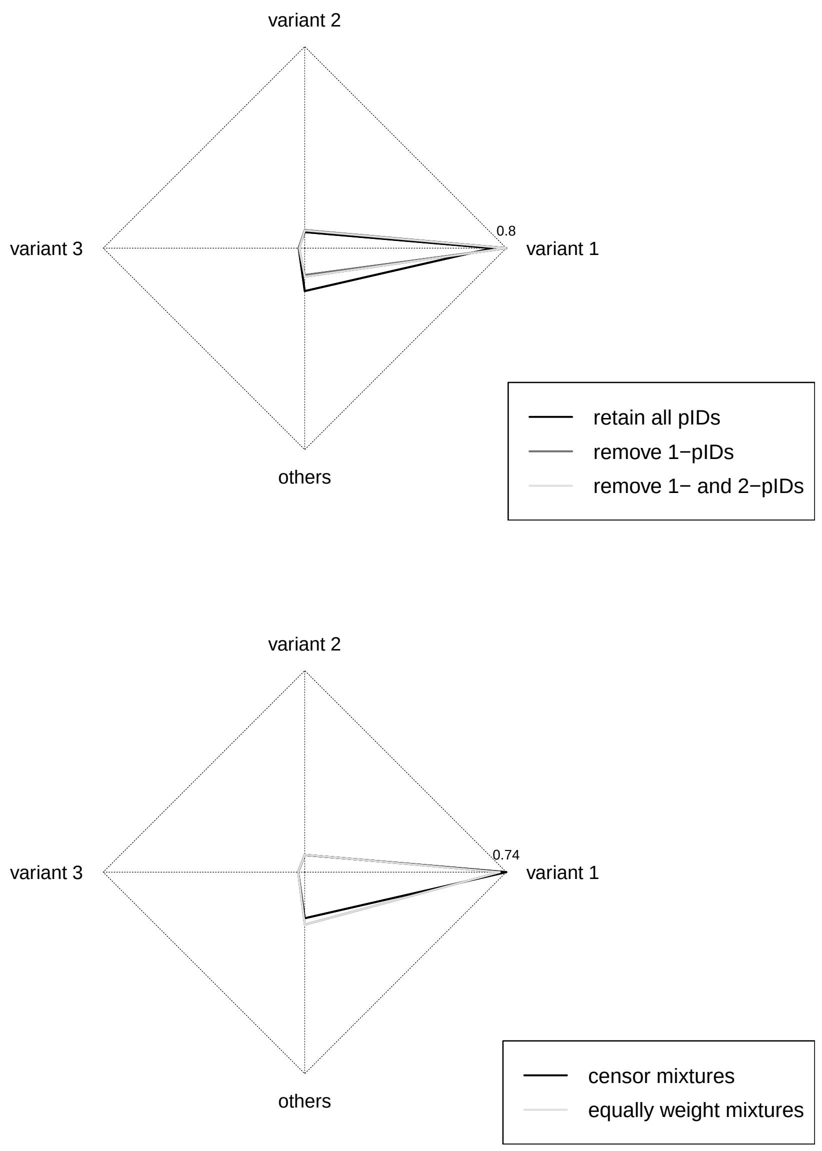

Figure S7: Analogous plots to those in figure 5 for the top three variants observed in subject P2. In the star plot of prevalences by keep scheme, a censoring mixture resolution scheme was used for all settings; in the star plot of prevalences by mixture resolution scheme, all pIDs were retained for all settings.

	Estimate	Std. Error	t value	$\mathbb{P}\{>\|t\|\}$
(Intercept)	98.17	2578	0.04	0.97
homopolymer score	184	219.9	0.84	0.40
C content	3267	681	4.80	< 0.0001
G content	2771	640.5	4.33	< 0.0001
T content	1539	560.8	2.74	0.0061

Table S16: Coefficients of fit of pID "success" for HCV data. The baseline case is for the pID AAAAAAAAA. The fit assumed a gamma distribution with inverse link function.

Regression coefficients

	(Intercept)	NNDNNHNNV	NNNNNNNNN	$\log_{10}$(dilution)
variant 1	0.07199	0.09327	0.1489	0.04234
variant 2	-0.5493	-0.09284	-0.0153	-0.09483
variant 3	-1.568	-0.04745	0.02903	0.1045
variant 4	-1.814	0.1067	-0.2587	0.03768
variant 5	-2.039	-0.07967	0.2257	-0.2204
variant 6	-2.452	-0.1872	-0.3122	-0.0143

Standard errors

	(Intercept)	NNDNNHNNV	NNNNNNNNN	$\log_{10}$(dilution)
variant 1	0.05531	0.06122	0.07871	0.04197
variant 2	0.06676	0.07482	0.09705	0.05465
variant 3	0.09509	0.106	0.1364	0.07131
variant 4	0.1064	0.1168	0.1646	0.08197
variant 5	0.1208	0.1362	0.1669	0.1041
variant 6	0.1434	0.1622	0.2252	0.1207

p-values

	(Intercept)	NNDNNHNNV	NNNNNNNNN	$\log_{10}$(dilution)
variant 1	0.09653	0.06383	0.02927	0.1565
variant 2	< 0.0001	0.1073	0.4374	0.04135
variant 3	< 0.0001	0.3272	0.4157	0.07134
variant 4	< 0.0001	0.1806	0.05801	0.3229
variant 5	< 0.0001	0.2793	0.08815	0.01711
variant 6	< 0.0001	0.1242	0.08276	0.4528

Table S17: Coefficients, standard errors, and p-values (coming from a z-statistic) of a multinomial logistic regression on the prevalences of the top six variants observed for subject P1. The intercept assumes that design NBDHVBDHV and an undiluted extract were used.

Regression coefficients

	(Intercept)	NNNNNNNNN	$\log_{10}$(dilution)
variant 1	1.5	0.03371	-0.1731
variant 2	-0.9459	0.05057	-0.2828
variant 3	-1.974	0.283	-0.7865

Standard errors

	(Intercept)	NNNNNNNNN	$\log_{10}$(dilution)
variant 1	0.07618	0.1048	0.08942
variant 2	0.1316	0.1809	0.1733
variant 3	0.2034	0.2694	0.3606

p-values

	(Intercept)	NNNNNNNNN	$\log_{10}$(dilution)
variant 1	< 0.0001	0.3738	0.02644
variant 2	< 0.0001	0.3899	0.05138
variant 3	< 0.0001	0.1467	0.0146

Table S18: Analogue of table S17 for the top three variants observed for subject P2. The intercept assumes that design NBDHVBDHV and an undiluted extract were used.

Regression coefficients

	(Intercept)	remove singletons	remove 1- and 2-pIDs
variant 1	0.1674	0.3324	0.2312
variant 2	-0.6339	0.2174	0.1216
variant 3	-1.561	0.3014	0.3554
variant 4	-1.771	0.2985	0.2508
variant 5	-2.1	0.1628	0.1173
variant 6	-2.619	0.3626	0.3284

Standard errors

	(Intercept)	remove singletons	remove 1- and 2-pIDs
variant 1	0.02398	0.0639	0.08568
variant 2	0.02998	0.07996	0.1082
variant 3	0.04237	0.108	0.1391
variant 4	0.04629	0.1177	0.1572
variant 5	0.05344	0.1421	0.1906
variant 6	0.06771	0.1662	0.2205

p-values

	(Intercept)	remove singletons	remove 1- and 2-pIDs
variant 1	< 0.0001	< 0.0001	0.003484
variant 2	< 0.0001	0.003273	0.1306
variant 3	< 0.0001	0.002632	0.005321
variant 4	0	0.005598	0.05534
variant 5	0	0.1259	0.2691
variant 6	0	0.01454	0.0682

Table S19: Coefficients, standard errors, and p-values (coming from a z-statistic) from a multinomial logistic regression on the prevalences of the top six variants observed in subject P1, using keep scheme as the explanatory factor. The intercept assumes that all pIDs were retained; in all settings, a censoring mixture resolution scheme was employed.

Regression coefficients

	(Intercept)	equal weighting
variant 1	0.1674	-0.05128
variant 2	-0.6339	-0.04073
variant 3	-1.561	-0.05536
variant 4	-1.771	-0.04104
variant 5	-2.1	-0.05014
variant 6	-2.619	-0.04343

Standard errors

	(Intercept)	equal weighting
variant 1	0.02398	0.03343
variant 2	0.02998	0.04182
variant 3	0.04237	0.05939
variant 4	0.04629	0.06471
variant 5	0.05344	0.07488
variant 6	0.06771	0.09478

p-values

	(Intercept)	equal weighting
variant 1	< 0.0001	0.0625
variant 2	< 0.0001	0.165
variant 3	< 0.0001	0.1756
variant 4	0	0.263
variant 5	0	0.2516
variant 6	0	0.3234

Table S20: Coefficients, standard errors, and p-values (coming from a z-statistic) of a multinomial logistic regression on the prevalences of the top six variants observed in subject P1, using mixture resolution scheme as the explanatory factor. The intercept corresponds to a censoring scheme; in both settings, all pIDs were retained.

Regression coefficients

	(Intercept)	remove singletons	remove 1- and 2-pIDs
variant 1	1.475	0.5365	0.4785
variant 2	-0.9838	0.5823	0.5084
variant 3	-1.955	0.4667	0.2898

Standard errors

	(Intercept)	remove singletons	remove 1- and 2-pIDs
variant 1	0.05214	0.1089	0.1347
variant 2	0.09016	0.1681	0.2083
variant 3	0.1336	0.2482	0.3206

p-values

	(Intercept)	remove singletons	remove 1- and 2-pIDs
variant 1	< 0.0001	< 0.0001	0.0001914
variant 2	< 0.0001	0.0002649	0.007329
variant 3	< 0.0001	0.03004	0.183

Table S21: Analogue of table S19 for the top three variants observed in subject P2.

Regression coefficients

	(Intercept)	equal weighting
variant 1	1.475	-0.1623
variant 2	-0.9841	-0.1214
variant 3	-1.953	-0.1238

Standard errors

	(Intercept)	equal weighting
variant 1	0.05214	0.0709
variant 2	0.09018	0.1243
variant 3	0.1335	0.1848

p-values

	(Intercept)	equal weighting
variant 1	< 0.0001	0.01105
variant 2	< 0.0001	0.1643
variant 3	< 0.0001	0.2515

Table S22: Analogue of table S20 for the top three variants observed in subject P2.
